# Absence of strong strain effects in behavioral analyses of *Shank3*-deficient mice

**DOI:** 10.1242/dmm.013821

**Published:** 2014-03-20

**Authors:** Elodie Drapeau, Nate P. Dorr, Gregory A. Elder, Joseph D. Buxbaum

**Affiliations:** 1 Seaver Autism Center for Research and Treatment, Icahn School of Medicine at Mount Sinai, New York, NY 10029, USA.; 2 Department of Psychiatry, Icahn School of Medicine at Mount Sinai, New York, NY 10029, USA.; 3 Friedman Brain Institute, Icahn School of Medicine at Mount Sinai, New York, NY 10029, USA.; 4 Department of Neurology, Icahn School of Medicine at Mount Sinai, One Gustave L. Levy Place, New York, NY 10029, USA.; 5 Neurology Service, James J. Peters VA Medical Center, Bronx, NY 10468, USA.; 6 Department of Pharmacology and Systems Therapeutics and Systems Biology Center New York, Icahn School of Medicine at Mount Sinai, New York, NY 10029, USA.; 7 Department of Genetics and Genomic Sciences, Icahn School of Medicine at Mount Sinai, New York, NY 10029, USA.; 8 Department of Neuroscience, Icahn School of Medicine at Mount Sinai, New York, NY 10029, USA.; 9 Mindich Child Health and Development Institute, Icahn School of Medicine at Mount Sinai, New York, NY 10029, USA.

**Keywords:** Shank3, Phelan-McDermid syndrome, Autism spectrum disorders, 22q13, Mouse strain, Genetic modifier, Behavior

## Abstract

Haploinsufficiency of *SHANK3*, caused by chromosomal abnormalities or mutations that disrupt one copy of the gene, leads to a neurodevelopmental syndrome called Phelan-McDermid syndrome, symptoms of which can include absent or delayed speech, intellectual disability, neurological changes and autism spectrum disorders. The SHANK3 protein forms a key structural part of the post-synaptic density. We previously generated and characterized mice with a targeted disruption of *Shank3* in which exons coding for the ankyrin-repeat domain were deleted and expression of full-length Shank3 was disrupted. We documented specific deficits in synaptic function and plasticity, along with reduced reciprocal social interactions, in *Shank3* heterozygous mice. Changes in phenotype owing to a mutation at a single locus are quite frequently modulated by other loci, most dramatically when the entire genetic background is changed. In mice, each strain of laboratory mouse represents a distinct genetic background and alterations in phenotype owing to gene knockout or transgenesis are frequently different across strains, which can lead to the identification of important modifier loci. We have investigated the effect of genetic background on phenotypes of *Shank3* heterozygous, knockout and wild-type mice, using C57BL/6, 129SVE and FVB/Ntac strain backgrounds. We focused on observable behaviors with the goal of carrying out subsequent analyses to identify modifier loci. Surprisingly, there were very modest strain effects over a large battery of analyses. These results indicate that behavioral phenotypes associated with *Shank3* haploinsufficiency are largely strain-independent.

## INTRODUCTION

SHANK3 is a synaptic scaffolding protein that forms a key structural part of the post-synaptic density of excitatory synapses (Kreienkamp, 2008), where it interacts with numerous other post-synaptic proteins, including Homer, ionotropic and metabotropic glutamate receptors, neuroligin, and components of the actin cytoskeleton (Boeckers et al., 1999; Naisbitt et al., 1999; Tu et al., 1999; Sheng and Kim, 2000; Boeckers, 2006; Kreienkamp, 2008; Betancur et al., 2009; Arons et al., 2012). *Shank3* overexpression promotes the formation of new synapses and dendritic spines and regulates their shape and size (Sala et al., 2001; Roussignol et al., 2005; Grabrucker et al., 2011; Verpelli et al., 2011; Arons et al., 2012; Durand et al., 2012), whereas *Shank3* knockdown or autism-associated *Shank3* mutations specifically impair glutamate signaling at synapses (Verpelli et al., 2011; Arons et al., 2012). Similarly, a decrease in glutamatergic transmission and long-term potentiation (LTP), neuronal hypertrophy and a reduction in spine density are observed in animal models of *Shank3* haploinsufficiency (Bozdagi et al., 2010; Peça et al., 2011; Wang et al., 2011; Yang et al., 2012).

In humans, haploinsufficiency of *SHANK3*, caused either by chromosomal abnormalities or mutations that disrupt one copy of the gene, leads to Phelan-McDermid syndrome (PMS), a neurodevelopmental syndrome that is characterized by global developmental delay, intellectual disability, delayed or absent speech, and autism spectrum disorder (ASD), as well as hypotonia and other motor deficits, seizures, minor dysmorphic features and additional medical issues (Wilson et al., 2003; Bonaglia et al., 2006; Cusmano-Ozog et al., 2007; Durand et al., 2007; Moessner et al., 2007; Phelan, 2008; Herbert, 2011; Soorya et al., 2013). PMS is both one of the most penetrant and one of the most common monogenic causes of autism, and recent studies have shown *SHANK3* deletion or mutation in up to 1.7% of individuals with ASD (Moessner et al., 2007; Guilmatre et al., 2009; Qiao et al., 2009; Sykes et al., 2009; Pinto et al., 2010; Rosenfeld et al., 2010; Schaefer et al., 2010; Shen et al., 2010; Bremer et al., 2011; Waga et al., 2011; Gong et al., 2012; Betancur and Buxbaum, 2013). However, despite common features, there is a significant variability in the expression and severity of the phenotype observed in individuals with PMS (Goizet et al., 2000; Manning et al., 2004; Cusmano-Ozog et al., 2007; Dhar et al., 2010; Phelan and Betancur, 2011; Soorya et al., 2013).

The creation of mice with a *Shank3* gene deletion has helped define the role of the protein in synaptic function and in downstream behavioral changes. Bozdagi et al. have reported the generation of a *Shank3*-deficient mouse with a targeted disruption in one copy of *Shank3* in which exons 4–9 (coding for the ankyrin repeat domain) were deleted and expression of full-length Shank3 was disrupted (Bozdagi et al., 2010). These mice display reduced expression of glutamate receptor 1 and show deficits in synaptic function and plasticity, specifically in LTP, in hippocampal CA1 pyramidal neurons. Male heterozygotes also show less social sniffing and emit fewer ultrasonic vocalizations during interactions with estrus female mice (Bozdagi et al., 2010). In a follow-up study, similar, but more severe, deficits were observed in knockout mice (Yang et al., 2012).

**TRANSLATIONAL IMPACT**

**Clinical issue**

Phelan-McDermid syndrome (PMS) is a rare genetic syndrome in which one copy of the q13 portion of chromosome 22 is missing or mutated, leading to a global developmental delay, delayed or absent speech, and autistic behaviors. Several genes map to the genomic region linked with the disease; however, there is overwhelming evidence that *SHANK3* –a gene encoding an essential protein for communication between neurons – causes the neurological and behavioral aspects of the syndrome. The effect of a gene on specific behaviors can be modified by other genes, and this effect can be dramatic when the entire genetic background is changed. Quantitative trait locus (QTL) analysis – a statistical method that links phenotypic data and genotypic data – can be used to identify genes that change the severity of a particular phenotype. The identification of modifier genes is a powerful means to dissect molecular pathophysiology, discover mechanisms underlying neurodevelopmental phenotypes and provide additional targets for drug development.

**Results**

To determine whether the broad range of impairments observed in PMS could be explained by modulation by other genetic loci, the authors generated *Shank3*-deficient mice using three different strains to provide distinct genetic backgrounds. They tested the animals using an extensive battery of behavioral tests designed to assess the main features of PMS. As in previous studies using similar or slightly different mouse models of *Shank3* deficiency, they observed altered phenotypes in different subcategories of behaviors. Surprisingly, however, there were very modest strain effects over a large battery of analysis: few tests revealed significant differences across the different strains or genetic backgrounds.

**Implications and future directions**

This study demonstrates that the genetic background is not a determining factor of the phenotype in *Shank3*-deficient mice and that quantitative trait locus analysis is unlikely to reveal the existence of genetic modifiers. Although this does not rule out the existence of a genetic modifier effect in humans, it suggests that variability observed between individuals is explained by a factor other than interactions with background genes. Recent studies have shown a correlation between the size of the deletion encompassing *Shank3* in individuals with PMS and the severity of the phenotype. Future analysis of the influence of the other genes affected by the deletion might contribute to our understanding of the molecular mechanisms involved in PMS.

In another mouse model with a similar deletion, knockout mice also display impaired LTP and altered dendritic spine morphology (Wang et al., 2011). These mice also show alterations in motor learning, vocalization, social behavior, repetitive behaviors, learning and memory. Peça et al. have generated *Shank3*-variant-deficient mice with a targeted disruption of the PDZ domain (Peça et al., 2011). They report altered cortico-striatal input-output functions, as well as changes in miniature excitatory post-synaptic current (mEPSC) frequency and amplitude in striatal medium spiny neurons. The behavioral characterization of these mice shows excessive grooming, along with altered responses in social approach and preference for social novelty paradigms (Peça et al., 2011).

Changes in synaptic plasticity and behavior caused by a mutation at a single locus are frequently modulated by other factors, including the prenatal and postnatal environment or by other genetic loci, which can be most easily demonstrated when the entire genetic background is changed. Each strain of laboratory mouse represents a distinct genetic background that is known to significantly alter baseline behaviors (Crawley et al., 1997; Holmes et al., 2002; Bothe et al., 2004; Moy et al., 2007), and several studies have demonstrated that the genetic background has a significant impact on multiple behavioral responses, including ASD-like traits, in mouse models of Fragile X syndrome, for example (Dobkin et al., 2000; Errijgers et al., 2008; Baker et al., 2010; Pietropaolo et al., 2011; Spencer et al., 2011). When such a change is identified, it can lead to identifying modifier loci that modulate phenotypes, as has recently been done in models for Rett syndrome (Buchovecky et al., 2013; Grillo et al., 2013), leading to a better understanding of pathophysiology and opportunities for therapeutics.

In PMS, there is some suggestion of genetic modifier loci. Mutation sizes are highly variable, ranging from point mutations to deletions covering tens of kilobases to over nine megabases. Several studies have shown correlations between the size of the deletion and the severity of some phenotypes (Wilson et al., 2003; Jeffries et al., 2005; Moessner et al., 2007; Phelan, 2008; Dhar et al., 2010; Bonaglia et al., 2011; Cooper et al., 2011; Sarasua et al., 2011; Soorya et al., 2013). In the current study, to investigate the role of genetic background on *Shank3*-deficient mice, animals that had been generated on the C57BL/6 (C57) background (Bozdagi et al., 2010; Yang et al., 2012) were backcrossed onto two additional genetic backgrounds, FVB/Ntac (FVB) and 129SVE. *Shank3* wild-type, heterozygous and knockout mice from the three genetic backgrounds were evaluated in a battery of tests that had been designed to assess the main features of PMS, including assays for neurodevelopment changes, sensory-motor defects, hyperactivity, emotionality and anxiety, as well as behaviors relevant to ASD.

## RESULTS

### General health

**Fig. 1.**
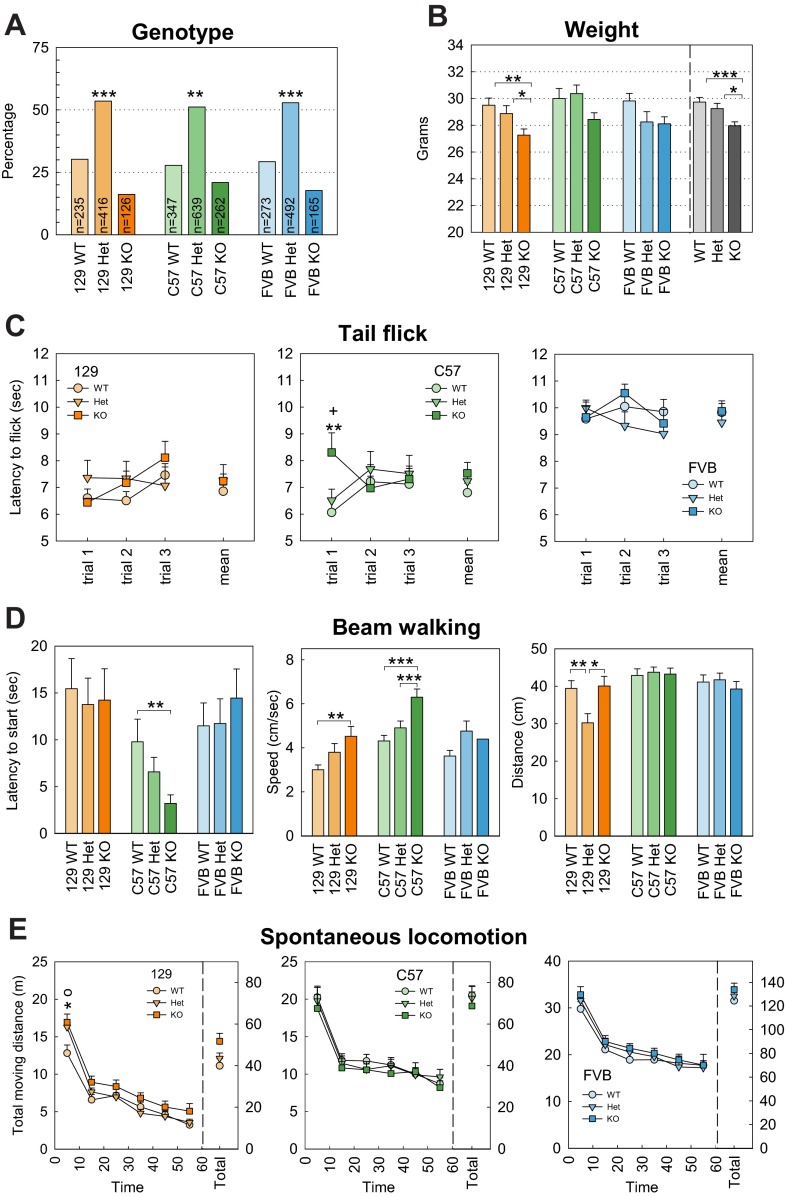
**General health and sensory-motor performances in *Shank3*-deficient mice.** (A) Distribution of genotype. A deficit in the number of knockout (KO) mice was observed in all strains. (B) Weight of mice. Knockout mice were significantly smaller than wild type (WT) and heterozygotes (Het) (the main effect of genotype across the three strains, *P*=0.001). 129, 129SVE. (C) Tail-flick assay. A higher tail-flick latency in response to a hot stimulus was observed during the first trial for the C57 strain knockout mice. (D) Beam walking. A significant decrease of the latency to the start of crossing the beam (left panel) and a shorter crossing time (middle panel) was observed in the C57 strain knockout mice. The distance traveled on the beam (right panel) was significantly shorter for 129SVE heterozygous mice, mainly because of an increase in the number of falls and freezing behavior; knockout animals on the 129SVE background performed similar to controls. (E) Spontaneous locomotion. The distance traveled over a 1-hour session in the open-field was similar for all genotypes; however, during the first ten minutes, a significant increase in locomotion was observed in 129SVE heterozygous and knockout animals. Data represents the means±s.e.m. *P<0.05, **P<0.01, ***P<0.001. In C and E: * WT versus KO, ^o^ WT versus Het, ^+^ Het vs KO.

Abnormal Mendelian ratios at the time of weaning were observed for the three strains, showing a significant deficit for *Shank3* knockout mice (Fig. 1A). For the 129SVE strain, out of 777 animals, 53.54% were *Shank3* heterozygotes, 30.25% were wild type and 16.22% were *Shank3* knockout (*P*=3.26×10^−8^). For the C57 strain, out of 1248 animals, 51.20% were heterozygotes, 27.80% were wild type and 21.00% were knockout (*P*=0.02). For the FVB strain, out of 930 animals, 52.90% were heterozygous, 23.35% were wild type and 17.71% were knockout (*P*=7.45×10^−7^).

The body weight at 3 months of age was similar for the three strains (129SVE: *n*=54, 28.56±0.32 g; C57: *n*=50, 29.59±0.37 g; FVB: *n*=51, 28.72±0.37 g; F_2,152_=2.037, *P*=0.134). However, for the 129SVE strain, the body weight of knockout mice was slightly lower than that of their wild-type and heterozygous littermates [Fig. 1B; wild type (WT): *n*=18, 29.50±0.54 g; heterozygote (Het): *n*=18, 28.89±0.58 g; knockout (KO): *n*=18, 27.28±0.46 g; F_2,51_=4.84, *P*=0.012; post-hoc: WT=Het, *P*=0.339; WT>KO, *P*=0.004; Het>KO, *P*=0.044]. A similar trend was observed for C57 mice (Fig. 1B; WT: *n*=12, 30.00±0.76 g; Het, *n*=20, 30.37±0.63 g; KO, *n*=18, 28.44±0.49 g; F_2,47_=2.86, *P*=0.068) and FVB strains (Fig. 1B; WT: *n*=17, 29.82±0.37 g; Het: *n*=16, 28.25±0.78 g; KO: *n*=18, 28.11±0.53 g; F_2,48_=2.34, *P*=0.107). A genotype and strain interaction showed an absence of strain effect (F_2,145_=2.30, *P*=0.104) or strain and genotype effect (F_2,145_=0.899, *P*=0.466), but there was a significant main effect of genotype across the three strains (F_4,145_=7.165, *P*=0.001). The body weight of knockout mice was significantly decreased compared with that of wild-type and heterozygous mice (WT: *n*=47, 29.74±0.34 g; Het: 29.25±0.39, KO: 27.97±0.29; post-hoc: WT=Het, *P*=0.236; WT>KO, *P*<0.001; Het>KO, *P*=0.011).

Because PMS is often as associated with medical conditions such as craniofacial abnormalities, dysmorphic features (hands and feet), abnormal spine curvature, microcephaly, renal, cardiac or respiratory problems, or lymphedema, three 2-month-old male mice from each strain and genotype were submitted to a comprehensive anatomic phenotyping that included gross and microscopic examination of tissues and organs. Three mice in the 129SVE background group exhibited testicular degeneration that was characterized by partial to complete loss of germinal cells in the seminiferous tubules and few multinucleated giant cells (data not shown). Epididymides that were associated with the affected testes were empty, or contained few round nucleated cells. However, testicular degeneration has been reported to occur spontaneously in 129SVE mice, and lesions were present in all three genotypes; hence, these lesions were not associated with *Shank3* deficiency. All mice on the FVB background exhibited marked diffuse bilateral outer-retinal degeneration with loss of the outer plexiform layer, outer nuclear layer and photoreceptors (data not shown). Mice on the FVB background are known to have early-onset retinal degeneration that is associated with the *rd* mutation. For this reason, before behavioral experiments, the visual acuity of our mice was tested using the visual placing test. All of the mice, including those on the FVB background, exhibited a visual placing reflex (100% success, all strains and genotypes), indicating that there was no problem with vision in any of the strains. No other notable microscopic lesions were observed, and no anatomic phenotype attributable to the *Shank3* mutation was found. Complete blood counts and a blood chemistry screen were also performed; however, no differences were observed between genotypes (supplementary material Table S1).

### Sensory-motor function

For all sensory-motor function assays, detailed results are reported in supplementary material Table S2. We first examined sensitivity to thermal stimulation using the tail-flick assay (Fig. 1C). No significant differences between the genotypes were observed for the latency to respond to the stimulus in the 129SVE and FVB strains. Only C57 *Shank3* knockout mice had a longer tail-flick latency and, thus, lower pain sensitivity than their heterozygous and wild-type littermates on the first trial (F_2,51_=4.24, *P*=0.021; post-hoc: WT=Het, *P*=0.579; WT<KO, *P*=0.009; Het<KO, *P*=0.028), although this difference was not apparent in the following trials.

In the grip strength test, no significant differences were observed between the genotypes in any of the three strains studied, for both the mean of all trials and the best performance. However, there was a decreasing trend in the mean forelimb grip strength observed in the 129SVE knockout animals compared with their wild-type and heterozygous littermates (F_2,51_=2.71, *P*=0.097; mean grip strength: WT: 127.86±2.87 g; Het: 128.84±2.13 g; KO: 119.72±3.46 g).

Motor learning was evaluated with the accelerating Rotarod test. A learning effect, characterized by an improvement of the performances (latency to fall) over the three trials, was observed for both the 129SVE and C57 strains (trial effect: C57: F_2,51_=7.001, *P*=0.001; FVB: F_2,51_=6.083, *P*=0.005), but not for the 129SVE strain. However, there was no significant interaction between the trial and genotype for any of the strains, and latencies to fall were similar for all the genotypes.

The beam walking test (Fig. 1D) was used to detect subtle deficits in fine motor coordination and balance that might not be detected by other motor tests. The C57 strain *Shank3* knockout mice had a shorter latency to start crossing than their wild-type and heterozygous littermates (WT, F_2,51_=3.37, *P*=0.036; post-hoc: WT=Het, *P*=0.178; WT>KO, *P*=0.003; Het>KO, *P*=0.059), as well as taking a shorter time to cross the beam (data not shown; F_2,51_=4.46, *P*=0.013; post-hoc: WT=Het, *P*=0.234; WT>KO, *P*=5.57×10^−5^; Het>KO, *P*=8.40×10^−4^), and, therefore, a faster speed (F_2,51_=9.62, *P*=0.0001; post-hoc: WT=Het, *P*=0.344; WT<KO, *P*=5.57×10^−5^; Het<KO, *P*=8.40×10^−4^) in the beam crossing test. In the 129SVE strain, fewer heterozygous animals were able to fully cross the beam (cross distance: F_2,51_=5.30, *P*=0.006; post-hoc: WT>Het, *P*=0.004; WT=KO, *P*=0.927; Het<KO, *P*=0.020), mainly because of an increased percentage of animals that stopped (froze) while crossing (F_2,51_=2.67, *P*=0.072; post-hoc: WT<Het, *P*=0.073; WT=KO, *P*=0.563; Het>KO, *P*=0.059) or that fell from the beam (F_2,51_=2.360, *P*=0.037; post-hoc: WT<Het, *P*=0.014; WT=KO, *P*=0.805; Het>KO, *P*=0.102). Knockout animals of the 129SVE background, however, had performances that were similar to wild type. Finally, no difference was found between the genotypes in the FVB strain.

Spontaneous locomotion was recorded in an open-field for 60 minutes. No genotype difference was observed for any of the strains for the total moving distance (129SVE: F_240,10_=2.15, *P*=0.127; C57: F_240,10_=0.19, *P*=0.827; FVB: F_240,10_=0.461, *P*=0.633) or time (129SVE: F_240,10_=2.15, *P*=0.127; C57: F_240,10_=0.19, *P*=0.827; FVB: F_240,10_=0.461, *P*=0.633) when considering the whole hour of testing. However, a significant interaction between the genotype and time interval was observed for the moving distance (F_240,10_=2.42, *P*=0.09) and time (F_240,10_=2.231, *P*=0.17) of the 129SVE strain when the test was divided into 10-minute intervals; the 129SVE heterozygous and knockout animals were hyperactive during the first 10 minutes of the test when compared with their wild-type littermates (distance: F_2,51_=4.67, *P*=0.014; WT<Het, *P*=0.011; WT<KO, *P*=0.015; Het=KO, *P*=0.739; time: F_2,51_=5.31, *P*=0.008; post-hoc: WT<Het, *P*=0.007; WT<KO, *P*=0.009; Het=KO, *P*=0.678). After 10 minutes, the spontaneous activity was reduced and the three groups were no longer different.

### Anxiety-related behaviors

**Fig. 2.**
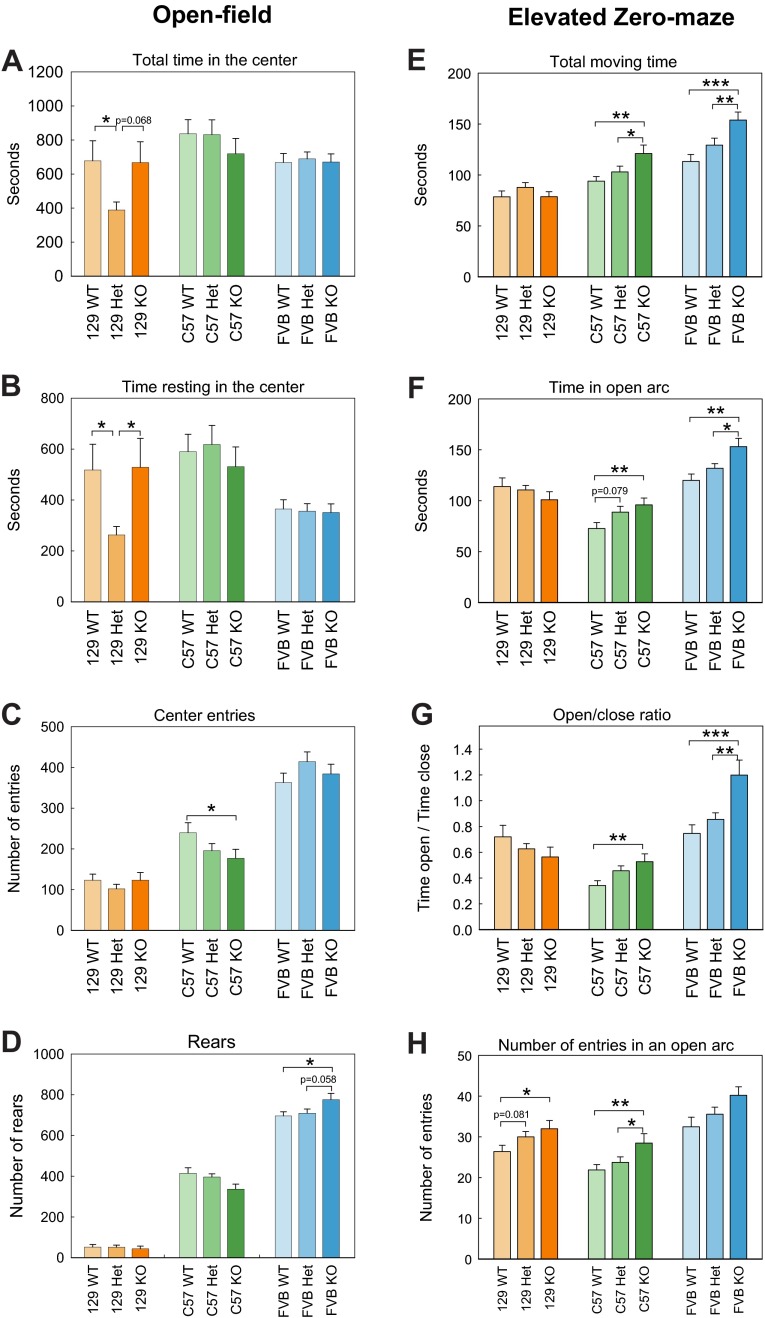
**Anxiety-like behavior in *Shank3*-deficient mice.** (A–D) Open-field. A decrease of thigmotaxis was observed in 129SVE (129) heterozygous (Het) and C57 knockout (KO) mice, as shown by a decrease of (A) the total time spent in the center, (B) the time spent resting in the center and (C) the number of times the mice entered into the central region. (D) An increase in the number of times the mice reared was observed for FVB knockout mice. (E–H) Elevated zero-maze. A reduction of anxiety characterized by an increase in (E) the total moving time, (F) the time spent in the open arcs and in the (G) open:closed arc ratio was observed in both the C57 and FVB *Shank3* mutant animals. (H) The number of entries into an open arc was increased in 129SVE and C57 knockout mice. Data represents means±s.e.m. *P<0.05, **P<0.01, ***P<0.001. WT, wild type.

Anxiety-like behaviors were observed in the open-field and in the elevated zero-maze, and detailed results are displayed in supplementary material Table S3. In the open-field (Fig. 2), mice were assessed for the distance traveled in the center, the total time spent in the center, the time spent resting in the center and the number of entries into the center. The number of times the mice reared, which is usually decreased in anxious mice, was also recorded. No significant difference between the genotypes of the FVB strain was observed in the thigmotaxis level over the 60-minute observation period (center distance: F_2,51_=0.55, *P*=0.582; center time: F_2,51_=0.05, *P*=0.949; entries into the center: F_2,51_=1.09, *P*=0.345; center rest: F_2,51_=0.06, *P*=0.944), but an increase in the number of times the mice reared was observed in the knockout animals (F_2,51_=3.05, *P*=0.05; post-hoc: WT=Het, *P*=0.654; WT>KO, *P*=0.025; Het=KO, *P*=0.058). No significant effects of an interaction between the time and genotype were observed for any of the parameters.

When compared with both the wild type and knockout, 129SVE *Shank3* heterozygous animals showed an increased level of thigmotaxis, which was characterized by a significant decrease in the total time the mice spent in the center (F_2,51_=3.26, *P*=0.047; post-hoc: WT>Het, *P*=0.024; WT=KO, *P*=0.923; Het=KO, *P*=0.068) and in the time spent resting in the center (F_2,51_=3.58, *P*=0.036; post-hoc: WT>Het, *P*=0.020; WT=KO, *P*=0.999; Het>KO, *P*=0.049). The distance that the mice traveled in the center, the number of center entries and the number of times the mice reared did not differ between the genotypes (distance: F_2,51_=0.58, *P*=0.564; center entries: F_2,51_=0.67, *P*=0.516; rears: F_2,51_=0.03, *P*=0.971). No significant interactions between the time and genotype were observed.

No significant differences in thigmotaxis levels were observed between the genotypes of the C57 strain over the 60-minute observation period (center time: F_2,51_=0.60, *P*=0.552; center rest: F_2,51_=0.39, *P*=0.676). However, a trend was found for the distance traveled in the center (F_2,51_=2.98, *P*=0.060) and the number of center entries (F_2,51_=2.43, *P*=0.099). Pairwise comparisons revealed that knockout animals traveled less in the center of the open-field (distance: WT=Het, *P*=0.125; WT>KO, *P*=0.020; Het=KO, *P*=0.354; number of entries: WT=Het, *P*=0.136; WT>KO, *P*=0.037; Het=KO, *P*=0.4804). Although the genotypes did not display differences over the cumulative 60-minute observation time, a significant interaction between the time and genotype was found for the time spent in the center, both for the overall (F_240,10_=2.57, *P*=0.006) and resting (F_240,10_=2.57, *P*=0.027) time. Pairwise comparisons revealed a significant increase in the time spent in the center during the last 10 minutes of the test for the C57 heterozygotes and a non-significant increase for the knockout mice compared with their wild-type littermates (F_2,51_=3.6, *P*=0.030; post-hoc: WT=Het, *P*=0.371; WT>KO, *P*=0.091; Het>KO, *P*=0.011), whereas the amount of time spent resting in the center was lower for knockout mice during the first 10 minutes of the test (F_2,51_=3.12, *P*=0.050; post-hoc: WT<Het, *P*=0.021; WT<KO, *P*=0.063; Het=KO, *P*=0.690). A significant difference between genotypes was also observed for the number of times the mice reared (F_2,51_=3.13, *P*=0.049), showing that there was a significant decrease in the number of times the knockout animals reared in comparison with wild-type mice, and comparison with heterozygous mice showed a close to significance *P*-value (WT=Het, *P*=0.319; WT>KO, *P*=0.021; Het=KO, *P*=0.065).

In the elevated zero-maze (Fig. 2), 129SVE *Shank3*-deficient and control animals performed at similar levels in all of the parameters studied, with the exception of the open arc entries, which were significantly increased in knockout animals, and a similar trend was observed in the heterozygous animals (F_2,51_=3.15, *P*=0.050; post-hoc: WT=Het, *P*=0.081; WT<KO, *P*=0.023; Het=KO, *P*=0.416). For both the C57 and FVB mouse strains, significant differences between the genotypes were observed in most of the parameters (the total moving distance and time, the time in the open and closed arcs, and the ratio of time spent in open or closed arcs). The knockout animals were more active (C57 total moving time: F_2,51_=4.40, *P*=0.018; post-hoc: WT=Het, *P*=0.439; WT<KO, *P*=0.006; Het<KO, *P*=0.030; C57 total moving distance: F_2,51_=7.29, *P*=0.002; WT=Het, *P*=0.532; WT<KO, *P*=0.001; Het<KO, *P*=0.003; FVB total moving time: F_2,51_=6.82, *P*=0.002; post-hoc: WT=Het, *P*=0.3444; WT<KO, *P*=0.001; Het<KO, *P*=0.009; FVB total moving distance: F_2,51_=6.63, *P*=0.003; WT=Het, *P*=0.372; WT<KO, *P*=0.001; Het<KO, *P*=0.010) and spent more time exploring the open arcs and less time in the closed arcs of the maze compared with wild-type and heterozygous animals (open:closed arc time ratio, C57: F_2,51_=4.37, *P*=0.018; post-hoc: WT=Het, *P*=0.096; WT<KO, *P*=0.005; Het=KO, *P*=0.157; FVB: F_2,51_=7.02, *P*=0.002; post-hoc: WT=Het, *P*=0.355; WT<KO, *P*=0.001; Het<KO, *P*=0.008). For the C57 strain mice, the number of entries into open arcs was also higher in knockout animals compared with wild-type and heterozygous animals (F_2,51_=5.36, *P*=0.008; post-hoc: WT=Het, *P*=0.532; WT<KO, *P*=0.004; Het<KO, *P*=0.012).

### Social behaviors

For all of the assays assessing social behaviors, detailed results are reported in supplementary material Table S4. In the test for social preference, a preference for the animal over the object was found in each strain for all genotypes, this was characterized by more time spent in the chamber containing the novel mouse than in the chamber containing the novel object (129SVE: WT: t_18_=2.38, *P*=0.029; Het: t_22_=4.27, *P*<0.001; KO: t_11_=4.22, *P*=0.001; C57: WT: t_16_=3.45, *P*=0.003; C57: Het: t_19_=3.038, *P*=0.007; KO: t_16_=5.34, *P*<0.001; FVB: WT: t_17_=7.19, *P*<0.001; Het: t_18_=8.54, *P*<0.001; KO: t_16_=5.72, *P*<0.001).

In the test for preference of social novelty and social recognition, the 129SVE mice failed to show a significant preference for the unfamiliar mouse compared with the familiar mouse; however, a trend was observed in knockout mice, which spent more time with the unfamiliar mouse than with the familiar mouse (129SVE: WT: t_18_=−1.30, *P*=0.210; Het, t_22_=−1.57, *P*=0.130; KO: t_11_=−1.99, *P*=0.071). By contrast, both the C57 and FVB strains showed a strong preference for the unfamiliar mouse, and mice of all genotypes spent significantly more time in the chamber containing the novel mouse than in the chamber containing the familiar mouse (C57: WT: t_16_=−4.71, *P*<0.001; Het: t_19_=−7.77, *P*<0.001; KO: t_16_=−7.78, *P*<0.001; FVB: WT: t_17_=−3.83, *P*<0.001; Het: t_18_=−4.07, *P*<0.001; KO: t_16_=−3.95, *P*<0.001).

**Fig. 3.**
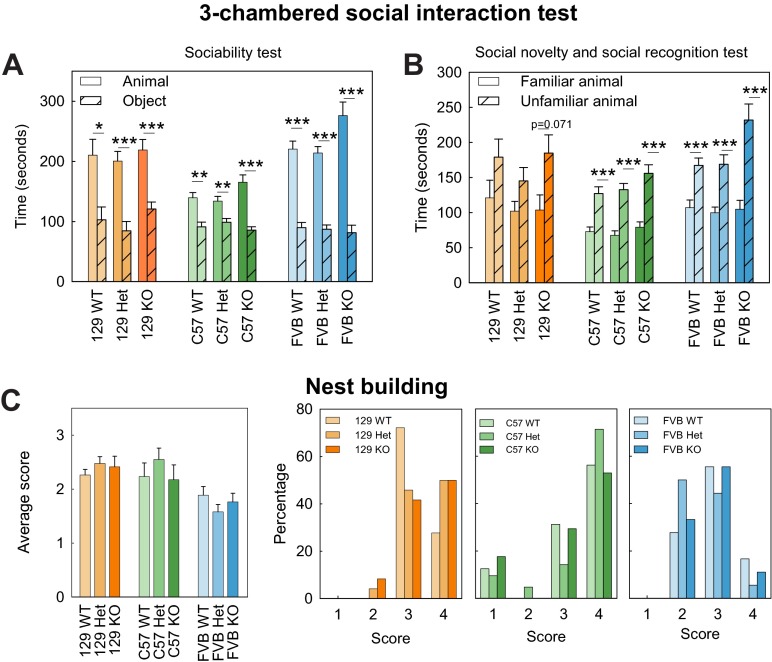
**Social behavior in *Shank3*-deficient mice.** (A,B) Three-chambered social approach test. Normal sociability, characterized by significantly more time spent with another mouse compared with an object, was found for mice from all genotypes and all strains in the sociability test (A). C57 and FVB mice spent significantly more time with the unfamiliar mouse in the social novelty and recognition test. The 129SVE (129) knockout (KO) mice showed a trend in their preference (B). (C) Nest building. No differences between the genotypes were found for any of the mouse strains in the nest building assay. The graph on the left shows the average score for the nests built for each genotype, the three graphs on the right shows more detailed analysis of this data. Data represents means±s.e.m. *P<0.05, **P<0.01, ***P<0.001. Het, heterozygous; WT, wild type.

Normal nest building skills were found in all three strains of *Shank3* mice for all genotypes (Fig. 3), whether comparing the mean score (129SVE: F_2,51_=0.89, *P*=0.417; C57: F_2,51_=0.66, *P*=0.519; FVB: F_2,51_=1.36, *P*=0.266) or the distribution of the scores (129SVE: *P*=0.346; C57: *P*=0.322; FVB: *P*=0.355).

### Learning and memory

**Fig. 4.**
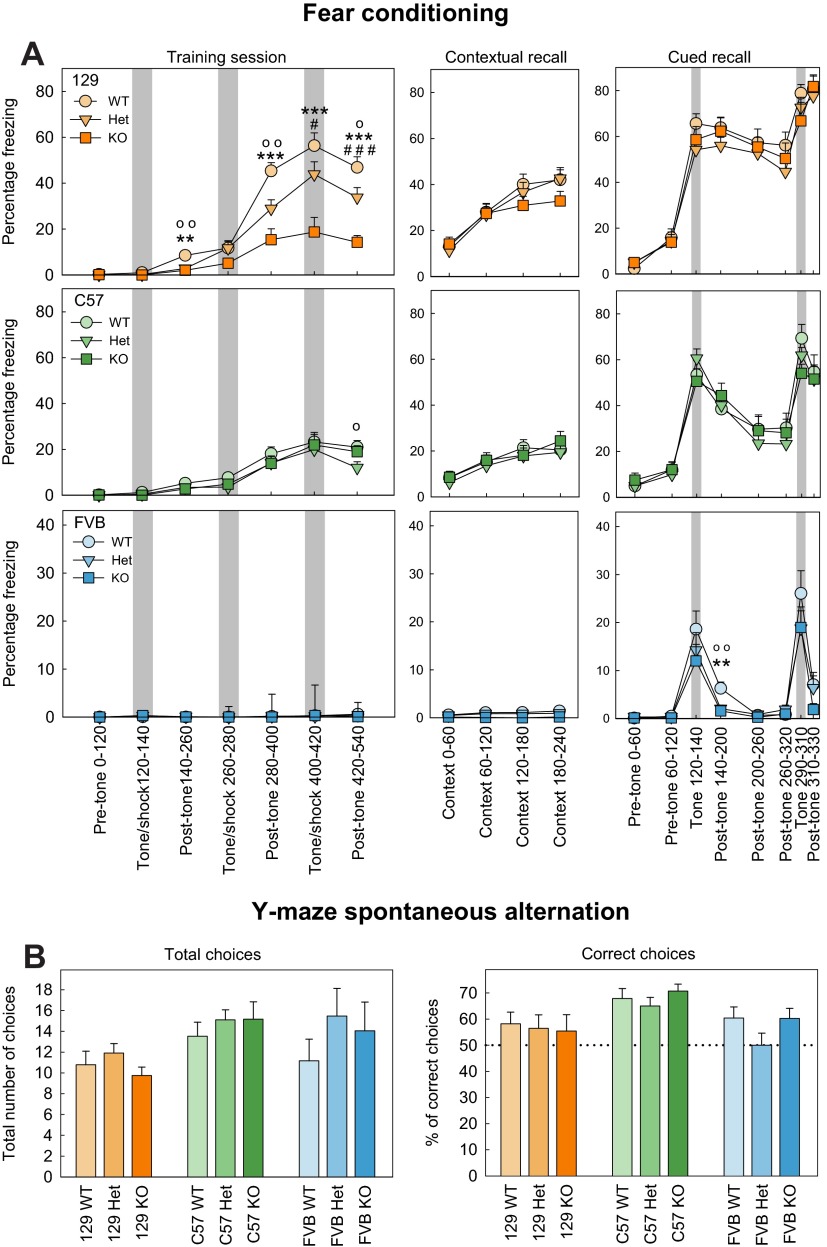
**Learning and memory in *Shank3*-deficient mice.** (A) Fear conditioning. Training (left column) was altered in *Shank3* heterozygous and knockout mice on the 129SVE (129) background, but no differences between the genotypes were found in either contextual (middle column) or cued recall (right column) phases. No differences between the genotypes were observed for C57 mice. All FVB mice were strongly impaired in all phases of the fear conditioning test. During cued recall, the percentage of mice that exhibited freezing behavior immediately after the first tone was lower in FVB *Shank3*-deficient animals than in wild-type. The *x*-axes indicate the time (minutes) for which the mice were recorded. (B) Y-maze spontaneous alternation. No differences were noted for the genotypes of 129SVE, C57 or FVB mice. The data represents means±s.e.m. ^o^*P*<0.05, ^oo^*P*<0.01, ^ooo^*P*<0.001 for wild-type (WT) versus heterozygous (Het) mice; *P<0.05, **P<0.01, ***P<0.001 for WT versus knockout (KO) mice; ^#^*P*<0.05, ^##^*P*<0.01, ^###^*P*<0.001 for Het versus KO mice.

The animals were tested in two learning and memory tests, specifically, fear conditioning and the Y-maze spontaneous alternation test, and detailed results are reported in supplementary material Table S5. Important differences were observed in fear conditioning for each of the strains (Fig. 4A). In the 129SVE strain, freezing behavior before presentations of tone-shock pairings was minimal and did not differ across the genotypes (F_2,51_=0.53, *P*=0.589), but significantly less freezing was observed in both heterozygous and knockout mice, compared with their wild-type littermates, in the periods following the three tone-shock associations and after the third association. Freezing was also lower in knockout compared with heterozygous animals (post-shock 1: F_2,51_=7.40, *P*=0.002; post-hoc: WT>Het, *P*=0.002; WT>KO, *P*=0.002; Het=KO, *P*=0.583; post-shock 2: F_2,51_=10.58, *P*=<0.001; post-hoc: WT>Het, *P*=0.003; WT>KO, *P*<0.001; Het=KO, *P*=0.065; post-shock 3: F_2,51_=13.44, *P*=<0.001; post-hoc: WT>Het, *P*=0.031; WT>KO, *P*<0.001; Het>KO, *P*<0.001). Surprisingly, no differences between the genotypes were found in the freezing behavior of the animals, neither during the contextual conditioning test (total freezing: F_2,51_=0.46, *P*=0.63) nor in the cued conditioning test (post-tone 1: F_2,51_=2.53, *P*=0.089; post-tone 2: F_2,51_=1.54, *P*=0.898). In the C57 strain, no differences in freezing behavior were found across the genotypes before presentations of tone-shock pairings (F_2,51_=0.41, *P*=0.664) or after the presentation of the two first tone-shock pairings during the training phase (post-shock 1: F_2,51_=1.25, *P*=0.295; post-shock 2: F_2,51_=0.60, *P*=0.552), but a decreasing trend in the percentage of heterozygous animals that exhibited freezing behavior was observed only after the last tone-shock pairing (post-shock 3: F_2,51_=3.14, *P*=0.052). Normal performances were observed for the C57 *Shank3*-deficient mice for both the contextual (total freezing: F_2,51_=0.21, *P*=0.810) and cued (post-tone 1: F_2,51_=0.20, *P*=0.815; post-tone 2: F_2,51_=0.10, *P*=0.904) fear conditioning sessions.

Because FVB mice are known to be visually impaired, special attention was given to use strong different sensorial cues (black and white stripes versus all white walls, fan on versus fan off, ethanol versus isopropanol scent, bar versus mesh grid floor). Despite these precautions, both training and recalls were strongly impaired in FVB mice compared with 129SVE or C57 mice, independent of the animal genotypes. FVB mice are generally very active animals and freezing behavior was not observed (freezing percentage<1.5%) for any of the genotypes during the training session or the contextual recall, showing an absence of short-term working memory and contextual learning. However, a temporary increase in freezing behavior was observed for the cued conditioning test during the two presentations of the tone. No differences were found on freezing during the tones between the genotypes (tone 1, F_2,51_=0.59, *P*=0.555; tone 2: F_2,51_=1.49, *P*=0.291). The percentage of mice that exhibited freezing behavior decreased immediately after the tones, but after the first tone this decrease was faster in heterozygous and knockout animals of the 129SVE strain compared with their wild-type littermates (F_2,51_=6.02, *P*=0.004; post-hoc: WT>Het, *P*=0.004; WT>KO, *P*=0.004; Het=KO, *P*=0.884).

In the Y-maze spontaneous alternation test (Fig. 4B), no differences were observed between the genotypes in any of the background strains regarding the total number of choices (129SVE: F_2,51_=0.45, *P*=0.585; C57: F_2,51_=0.48, *P*=0.617; FVB: F_2,51_=0.36, *P*=0.696) or the ratio of correct:total choices (129SVE: F_2,51_=0.08, *P*=0.927; C57: F_2,51_=0.59, *P*=0.560; FVB: F_2,51_=1.84, *P*=0.176) when compared by using ANCOVA. A Student’s *t*-test was then used to compare the score of each group with the probability of this occurring by chance (a ‘chance level’ of 50%). For all of the genotypes, C57 mice scored significantly above the chance level (WT: t_14_=4.74, *P*<0.001; Het: t_18_=4.64, *P*<0.001; KO: t_13_=−7.69, *P*<0.001), whereas, for all genotypes, 129SVE mice were not different from the chance level [although a trend was observed in the wild-type group (WT: t_13_=1.84, *P*=0.089; Het: t_19_=1.25, *P*=0.23; KO: t_8_=−0.012, *P*=0.99)]. For the FVB strain, both wild-type and homozygous mice were significantly above the chance level, whereas the heterozygous animals were not different from the chance level (WT: t_9_=2.44, *P*=0.037; Het: t_13_=0.00, *P*=1; KO: t_11_=−2.65, *P*=0.02).

### Repetitive and stereotypical behaviors

**Fig. 5.**
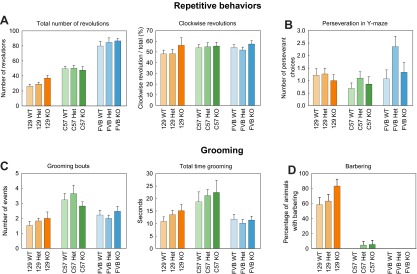
**Stereotypies, repetitive behavior and perseverance in *Shank3*-deficient mice.** (A,B) Repetitive behaviors. No significant differences were observed between the genotypes for (A) the number of revolutions in the open-field (the left panel shows the total number of revolutions, and the right panel shows the percentage of the total revolutions that were performed in a clockwise direction) or (B) perseveration (the number of times an animal choose the same arm of the maze for three consecutive times) in the Y-maze. (C,D) Grooming and barbering. No significant differences between the genotypes were observed for (C) the number of grooming bouts (left panel) or time (right panel) and (D) the percentage of animals with barbering. The data represent means±s.e.m. 129, 129SVE.

Repetitive behaviors are one of the core features of ASD. Therefore, during all of the behavioral tests, mice were also carefully monitored for stereotypies, including circling, as well as perseverative and repetitive behaviors, such as repetitive long episodes of self-grooming. Detailed results are reported in supplementary material Table S6. Circling was observed during the open-field test, and both clockwise and counterclockwise revolutions were scored (Fig. 5A). A trend towards an increase in the total number of revolutions was observed in the 129SVE strain knockout mice compared with their wild-type and heterozygous littermates (F_2,51_=2.52, *P*=0.091). A non-significant preference for clockwise revolutions was also observed for knockout mice. No difference was observed in the genotypes for both the C57 (total number of revolutions: F_2,51_=0.11, *P*=0.892, percentage of clockwise revolutions: F_2,51_=0.05, *P*=0.950) and FVB (total number of revolutions: F_2,51_=0.55, *P*=0.547, percentage of clockwise revolutions: F_2,51_=0.98, *P*=0.384) strains.

Because individuals with an ASD-like presentation can maintain rigid habits and frequently show a strong insistence on sameness and are upset by change in routine, the Y-maze spontaneous alternation test was used to evaluate perseveration by monitoring the resistance to the change of arm choice (Fig. 5B). A choice was considered perseverative when an animal re-entered the same arm of the maze that it had visited previously for three consecutive times. No significant difference across the genotypes was observed in the number of perseverative choices for any of the strains (129SVE: F_2,51_=0.10, *P*=0.907; C57: F_2,51_=0.61, *P*=0.549; FVB: F_2,51_=2.40, *P*=0.104). However, a trend towards making more perseverative choices was observed in FVB heterozygous animals compared with wild-type, but not knockout, mice (WT: 1.07±0.35, Het: 2.36±0.41, KO: 1.33±0.39).

Grooming was manually scored from the first 10 minutes of the open-field test, and the latency to the first grooming event, the number of grooming bouts and the total grooming time were measured (Fig. 5C). No significant difference between the genotypes was observed for any of the parameters on any of the strain backgrounds (latency: 129SVE: F_2,51_=0.67, *P*=0.517; C57: F_2,51_=0.60, *P*=0.552; FVB: F_2,51_=0.06, *P*=0.939; total number of bouts: 129SVE: F_2,51_=0.55, *P*=0.579; C57: F_2,51_=0.89, *P*=0.418; FVB: F_2,51_=0.69, *P*=0.506; total grooming time: 129SVE: F_2,51_=1.15, *P*=0.326; C57: F_2,51_=0.25, *P*=0.780; FVB: F_2,51_=0.50, *P*=0.785). However, for both the 129SVE and C57 strains of mice, a trend towards a progressive increase in the total time of grooming was observed, heterozygotes groomed for longer periods of time than wild-type animals, and knockout mice groomed for longer periods of time than heterozygotes (129SVE: WT: 10.84±1.83 seconds; Het: 13.57±0.18 seconds, KO: 15.17±2.44 seconds; C57, WT: 18.71±3.95 seconds, Het: 21.20±2.41 seconds, KO: 22.47±4.77 seconds). For the FVB strain, the total duration of grooming was similar for the three genotypes (WT: 11.78±1.86 seconds, Het: 10.16±1.54 seconds, KO: 11.41±1.55 seconds). Barbering was also observed in 129SVE strain mice that had been group-housed before the beginning of the behavioral testing, and this was scored after up to 8 weeks of social isolation, as well as in an additional cohort of animals (Fig. 5D; 129SVE: WT *n*=29; Het *n*=30; KO *n*=18; C57: WT *n*=31; Het *n*=41; KO *n*=35; FVB: WT *n*=35; Het *n*=35; KO *n*=35). In group-housed animals, the barbering was characterized by whisker removal, nasal alopecia and snout denuding, with bald patches found on the head, neck and body. In single-housed animals, barbering was mainly localized on the body (base of the tail, ventral area and inside of thighs), even if some head barbering did persist. This indicates that this behavior is likely to be, at least partially, self-initiated. Barbering was mostly specific to the 129SVE strain of mice; it was never observed in FVB mice and only in two C57 mice (one heterozygous, one knockout). In 129SVE mice, a suggestion of an increase in barbering was noted in knockout animals compared with wild type and heterozygotes (F_2,76_=1.61, *P*=0.206).

## DISCUSSION

In the present study, we have assessed the effects of three genetic backgrounds – C57BL/6, 129SVE and FVB – on the phenotype of *Shank3* wild-type, heterozygous and knockout mice in behaviors that are associated with PMS. We focused on sensory-motor defects, hyperactivity, emotionality and anxiety-like behaviors, cognitive abilities and additional mouse behaviors that are relevant to ASD – such as social, repetitive and perseverative behaviors. We observed moderate strain differences.

Independent of the genetic background, *Shank3* heterozygous and knockout mice did not display any gross or fine anatomical or histological abnormalities compared with wild-type littermates, even if known strain-related defects were reported. However, the number of knockout mice at the age of weaning was significantly lower than expected by Mendelian genetics, suggesting that there is a higher gestational or perinatal mortality rate. A decrease of weight at the age of 3 months was also observed for knockout animals. In individuals with PMS, accelerated growth is often observed; however, about 10% of affected children are small for their age, whereas some show growth beyond the 95th percentile (Prasad et al., 2000; Havens et al., 2004; Manning et al., 2004; Cusmano-Ozog et al., 2007; Phelan, 2008).

Hypersensitivity and hyposensitivity to sensory stimuli are frequently observed in individuals with PMS or ASD patients (Philippe et al., 2008; Phelan and Betancur, 2011). No sensory deficits have been reported in previous studies where animals were tested for olfaction, audition, vision, neuromuscular reflexes and pain sensitivity (Wang et al., 2011; Yang et al., 2012). In the current study, we observed that C57 *Shank3* knockout mice were less sensitive to pain, as measured by a higher latency in the tail-flick assay, but this was only observed in the first trial of the test and not in the two consecutive trials.

Hypotonia and motor learning difficulties have been reported in individuals with PMS and in some individuals with autism (Ghaziuddin and Butler, 1998; Rinehart et al., 2001; Moruzzi et al., 2011). Previous studies have shown that motor performances are impaired in *Shank3*-deficient mice in Rotarod, vertical pole and foot misplacement tests (Wang et al., 2011; Yang et al., 2012), as well as a slower speed in both the foot misplacement and open-field tests (Wang et al., 2011), although the Rotarod finding was not replicated in a third study (Peça et al., 2011). In the present study, we found no genotype-linked differences in the Rotarod or grip strength test, but the beam walking test revealed subtle differences. The distance traveled on the beam was significantly shorter for 129SVE heterozygous mice, mainly because of an increase in the number of falls and in freezing behavior; however, knockout animals were not impaired and had performances similar to controls.

Hyperactivity and anxiety are other common features of PMS (Goizet et al., 2000; Manning et al., 2004; Cusmano-Ozog et al., 2007; Philippe et al., 2008; Dhar et al., 2010; Sarasua et al., 2011; Soorya et al., 2013). No, or little, decrease in spontaneous locomotion in the open-field test has been reported previously for *Shank3*-deficient mice (Peça et al., 2011; Wang et al., 2011; Yang et al., 2012). We did not observe any differences in the distance that the mice traveled over a 1-hour session. However, during the first 10 minutes of observations, a significant increase in locomotion was observed for 129SVE heterozygous and knockout animals. An increase in the time and distance of movement was also observed for *Shank3*-deficient mice in the C57 and FVB strains in the zero-maze test, and an increased number of open arc entries was observed for the 129SVE and C57 strains. In addition, in the beam walking test, a significant decrease in the latency to start crossing was observed in the C57 knockout mice, and an increased speed was found for both 129SVE and C57 animals. Altogether, these data indicate a degree of hyperactivity in *Shank3*-deficient mice. Anxiety-like behavior was observed in the open-field and elevated zero-maze tests. As has been reported previously, thigmotaxis was found not to be affected by genotype (Peça et al., 2011; Wang et al., 2011; Yang et al., 2012), with the exception of the 129SVE heterozygous animals, which spent less time than both their wild-type and knockout littermates in the center of the open-field. Vertical activity was increased in *Shank3*-deficient animals of the FVB strain. In the zero-maze, a reduction in the level of anxiety, characterized by a significant increase in the time that was spent in the open arcs, was observed in both C57 and FVB *Shank3* mutant animals, whereas no difference (Wang et al., 2011; Yang et al., 2012) or a decrease (Peça et al., 2011) has been reported previously.

Impairment of social interactions, communication and stereotypic behaviors are the three core characteristics of ASD and have been reported, with variability, in some of the previous studies of PMS mouse models (Peça et al., 2011; Wang et al., 2011; Yang et al., 2012). A decrease in interactions between males and females (social sniffing and ultrasonic vocalization) has been observed in *Shank3*-deficient mice (Bozdagi et al., 2010), whereas no difference, or a trend towards a decrease in the interactions, have been reported in two different cohorts of the same *Shank3*-deficiency model (Yang et al., 2012). In the freely interacting dyadic test, decreased interaction was observed for knockout animals (Peça et al., 2011; Wang et al., 2011; Yang et al., 2012), whereas, in a sociability test, knockout animals failed to show a preference for a social stimulus when given the choice between social and non-social stimuli (Wang et al., 2011). Juvenile social interaction has also been shown to be altered in *Shank3*-deficient animals, even if the patterns of reduced social parameters were not identical in the two cohorts examined (Yang et al., 2012). In the three-chambered social approach test, no differences between the genotypes were reported by Yang and colleagues (Yang et al., 2012), whereas a lack of preference for an unfamiliar mouse over a familiar mouse was observed by Peça and colleagues (Peça et al., 2011). In the present study, all C57 and FVB animals spent a significantly longer amount of time with another mouse than with an object, or with an unfamiliar mouse than with a familiar one. In 129SVE animals, the time spent with another mouse over an object was significant, but only a trend was seen for an unfamiliar mouse versus a familiar one. The differences observed between cohorts of animals with the same, or similar, alteration of the *Shank3* gene can be explained by different factors, such as the age of the animals, the age of the mother and early handling of the pups (Yang et al., 2012). The social isolation because of the single-housing of the mice in our experiment is another stressful event that could explain the differences observed in several behavioral assays between our study and previous reports. No significant differences were found for stereotypies between the mouse genotypes. However, several trends were observed: the number of both the total revolutions and clockwise revolutions was increased in the 129SVE knockout animals, perseveration in the Y-maze was observed in FVB heterozygous mice and an increase in grooming was found for *Shank3*-deficient mice of both the 129SVE and C57 strains. Barbering is an abnormal repetitive behavior, which is analogous to human compulsive hair pulling, that is commonly observed in some strains of mice such as C57- and 129SVE-derived strains (Sarna et al., 2000; Kurien et al., 2005; Kalueff et al., 2006; Kalueff et al., 2007; Tynes, 2013). In the present study, barbering was observed in 66% of the 129SVE animals, but only in two mice of the C57 strain and none of the FVB animals. In the group-housed 129SVE animals, the barbering was characterized by whisker removal, nasal alopecia and snout denuding, with bald patches on the head, neck and body; this pattern is typical of the 129SVE strain, suggesting that hetero-barbering occurs between the mice (Kalueff et al., 2006). Animals were isolated for up to 8 weeks to allow the whiskers to regrow, because this could interfere with behavioral results; however, social isolation did not fully suppress the barbering, and a persistence of body barbering was still observed, although head barbering was attenuated but still present, which is consistent with self-barbering in 129SVE mice. Self-barbering has not been studied in 129SVE-derived mice, but it has been reported previously in both single- and group-housed male C57 animals (Garner et al., 2004; Garner et al., 2004b). Moreover, an increase in barbering was found in 129SVE knockout animals compared with their wild-type or heterozygote littermates.

The animals were tested in two learning and memory tests. Previous studies had found differing results in several learning and memory tests, such as the Morris water maze, the novel object recognition test and the social transmission of food preference (Wang et al., 2011; Yang et al., 2012). Here, we did not observe differences between the genotypes of any strain of mouse in the Y-maze spontaneous alternation test when the genotypes were compared by ANCOVA. Further analyses were used to compare each group to the chance level. The score of all of the genotypes of C57 mice were significantly above the chance level, whereas the choices of 129SVE mice were not too different to that expected if the decisions were occurring by chance. For the FVB strains, both the wild-type and knockout groups exhibited behavior that was significantly above the probability that this was occurring by chance, whereas the heterozygote group was not different from chance. In studies that have previously compared the strains, 129SVE-derived strains were shown to be impaired in the Y-maze spontaneous alternation task, with scores that were close to the chance level and significantly below that of C57 mice (Gerlai, 1998). Despite being visually impaired, the FVB strain is not known to be compromised in this test and obtains a score that is similar to that of the C57 strain (Farley et al., 2011). It should also be noted that the Y-maze spontaneous alternation test is an assay that is highly sensitive to the environment and that results can be affected by a number of parameters, such as the visual cues used, the room brightness and the stress of the animals (Hughes, 2004). In our protocol, animals were allowed to return to the starting arm on their own in order to avoid inducing stress through handling. In the fear conditioning paradigm, we observed a strong learning impairment for knockout and heterozygous animals of the 129SVE strain during the training phase, even if those animals performed similar to their wild-type littermates during the contextual and cued tests. All FVB animals performed poorly during the training and contextual recall but were able to show a moderate response to the tone during the cued test, even when the *Shank3* mutants were compared to wild-type littermates. Similar results have been reported in previous studies, all of which showed a lower percentage of mice that froze in all of the phases of fear conditioning in FVB-derived strains compared with other strains (Crawley et al., 1997; Owen et al., 1997; Bolivar et al., 2001; Bothe et al., 2005). More specifically, in a study that compared 12 inbred strains and seven F1 hybrid mice, FVB mice were shown to have the lowest level of baseline freezing (less than 3% of the total time), as well as significantly lower freezing responses to context (13% of the total time) compared with all of the other strains. Similar to what is observed in the current study, FVB mice have been shown to still be capable of freezing in response to the sound stimulus during the cued test (Owen et al., 1997). Here, normal fear conditioning was observed in all C57 mice genotypes.

The purpose of this study was to investigate the role of the genetic background on *Shank3*-deficient mice and to begin to determine if the broad range of impairments that are observed in PMS could be, at least in part, explained by modulation by other genetic loci. As in previous studies that used similar or slightly different mouse models of *Shank3* deficiency, we observed alterations in phenotypes in different subcategories of behaviors. Surprisingly, there were very modest strain effects over a large battery of analyses. Few tests showed significant strain differences (summarized in Table 1). For instance, an increase of the tail-flick latency in the first trial of the tail-flick test was observed in *Shank3* knockout C57 mice. In the beam walking test, a significant decrease in the latency to the start of crossing the beam, and a decrease in the crossing time was observed in the C57 strain knockout mice, and the distance traveled on the beam was significantly shorter for 129SVE heterozygous mice, mainly because of an increased number of falls and freezing behavior. In the open-field test, a significant increase in locomotion was observed in 129SVE heterozygous and knockout animals during the first 10 minutes of the test, and a decrease of thigmotaxis was observed in 129SVE heterozygotes and C57 strain knockout mice, whereas an increase in the number of times the mice reared was observed for FVB strain knockout mice. In the elevated zero-maze, a reduction of anxiety was observed in both the C57 and FVB strain *Shank3* mutant animals, and the number of entries into an open arc was increased in 129SVE and C57 knockout mice. Finally, in the fear conditioning test, the training was altered in 129SVE *Shank3* heterozygous and knockout mice. This demonstrates that the genetic background is not a determining factor of the phenotype in *Shank3*-deficient mice and that the variability observed between individual mice, even in different cohorts from the same strains (Bozdagi et al., 2010; Yang et al., 2012; present study), is not explained by interactions with background genes.

**Table 1.**
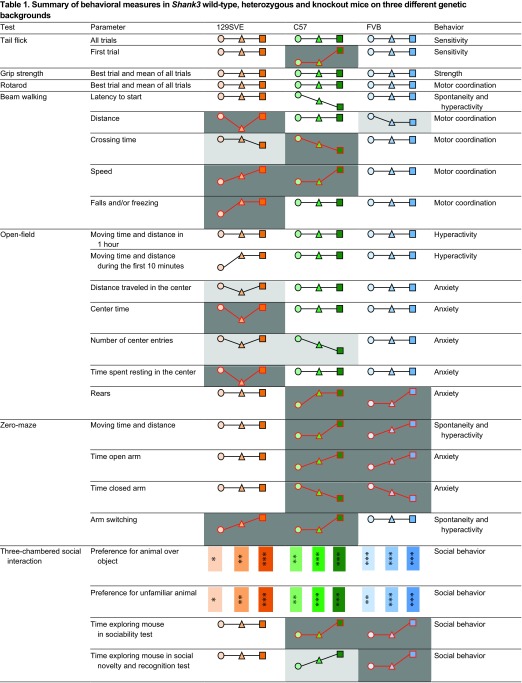

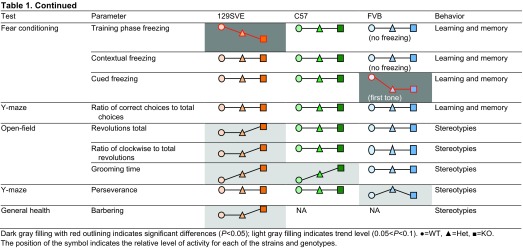
Summary of behavioral measures in *Shank3* wild-type, heterozygous and knockout mice on three different genetic backgrounds

## MATERIALS AND METHODS

### Generation of inbred strains of *Shank3*-deficient animals

All animal procedures were approved by the Institutional Animal Care and Use Committee of the Icahn School of Medicine at Mount Sinai and the James J. Peters Veterans Affairs Medical Center (JPVAMC). *Shank3*-deficient mice were generated using Bruce4 C57BL/6 embryonic stem cells, genotyped as described previously (Bozdagi et al., 2010; Yang et al., 2012) and maintained on a pure C57BL/6N background (Taconic, Germantown, NY). Two additional lines of mice were generated by backcrossing C57BL/6N animals on FVB/NTac and 129S6/SvEvTac (Taconic) backgrounds using the Taconic Speed Congenics program to achieve >99% congenic animals. For each line, heterozygotes were mated to generate litters that consisted of three genotypes – wild type, heterozygotes and knockout. The mice were weaned at 21 days of age, and one or two littermates from each genotype were group housed in standard plastic cages of three to six littermates per cage. Standard rodent chow and tap water were available *ad libitum*. The colony room was maintained on a 12 hours light-dark cycle at a constant temperature of 21–22°C and 55% humidity.

### Pathology

Comprehensive anatomic phenotyping of *Shank3*-deficient mice was performed by the Comparative Pathology Laboratory at Mount Sinai. The tissues that were examined included coronal sections of the entire head (brain, pituitary gland, nasal turbinates, teeth, tongue, skin, bone, eyes, ear), the spinal cord and vertebral column, heart, lungs, thymus, liver, gallbladder, spleen, kidneys, adrenal glands, salivary glands, cervical lymph nodes, esophagus, trachea, thyroid, skin, testes and epididymis, seminal vesicles, coagulating gland, prostate, prepucial glands, urinary bladder, pancreas, mesenteric lymph node, stomach, duodenum, jejunum, ileum, cecum, colon, skeletal muscle (thigh and diaphragm), stifle joint with bone, bone marrow, peripheral nerve, paw, sternum, white adipose and brown adipose tissue. A complete blood count, and a comprehensive chemistry screen was also performed.

### Behavioral testing

Behavioral testing was performed in the Mouse and Rat Phenotyping Shared Research Facility located at the JPVAMC. Animals were transferred to the facility at 7–9 weeks of age, single-housed and allowed a four-week acclimation period before testing. All testing was performed over a 4-week period by the same investigator who was blind to the genotypes in the order displayed below. Fear conditioning was conducted last to avoid this stressful task impacting on other behaviors. For each strain of mouse, 54 male animals were tested (129SVE: WT *n*=19, Het *n*=23, KO *n*=12; C57: WT *n*=17, Het *n*=20, KO *n*=17; FVB: WT *n*=18, Het *n*=19, KO *n*=17).

#### Visual acuity

Visual acuity was tested by using the visual placing test that takes advantage of the forepaw-reaching reflex: the mouse was held by its tail approximately 20 cm above the surface and was then progressively lowered. As it approaches the surface, the mouse should expand its forepaws to reach the floor. The test was repeated three times with a 30-second interval, and the forepaw-reaching reflex was quantified as the percentage of forepaw-reaching episodes that did not involve the vibrissae and/or nose touching the surface before the forepaws.

#### Tail-flick test

The tail-flick test was used to assess the nociceptive threshold. Awake mice were placed on a platform with their tail sitting on the groove of a heating panel. When the mice were calm, a narrow heat-producing beam was directed at a small discrete spot approximately 15 mm from the tip of the tail. The test was repeated three times with a 3-minute interval between each trial. The latency of the mice to flick their tail was recorded.

#### Grip strength

Forelimb muscle strength and function was evaluated with a Strength Meter (San Diego Instruments). This test relies on the instinctive tendency of mice to grasp an object with their forelimbs. The animal was pulled backwards gently by the tail while it grasped a pull bar that was connected to a tension meter. The force at the moment when the mouse lost its grip was recorded as the peak tension. The test was repeated three times with a 3-minute interval between trials. The mean of three trials, and the largest value from all trials, were used as parameters.

#### Rotarod

Motor coordination and endurance was assessed by using the Rotarod test (Harcard Apparatus). The mice were placed on an elevated accelerating rod (3-cm diameter) for three trials with a 15-minute interval between trials to avoid fatigue. Each trial lasted for a maximum of 2 minutes, during which the Rotarod undergoes a linear acceleration from 0 to 50 r.p.m. The animals were scored for their latency to fall.

#### Beam walking

Fine motor coordination and balance was assessed by the beam walking assay in which the mouse had to walk across an elevated horizontal beam (48-cm long, 2.5-cm diameter, 1-m above bedding) to a safe platform. Subjects were placed near one end in bright light, and the far end of the platform was placed in darkness, providing motivation to cross. The performance was quantified by measuring the latency to start crossing, the time to reach the platform or fall, the total distance that the mouse traveled and the number of paw slips and false starts (returns to the starting point). We used a 2-minute cut-off, and mice that successfully crossed the beam were given up to four additional trials with a 30-second interval between trials.

#### Open-field

Mice were tested in an automated open-field that was virtually divided into central and peripheral regions. Each mouse was allowed to explore the apparatus for 60 minutes. The distance traveled, the number of times that the mouse reared, the number of revolutions, the number of grooming bouts and the cumulative grooming time, the number of entries into the central region, and the time spent in the central and peripheral regions was recorded. Measures were recorded in 10-minute intervals.

#### Elevated zero-maze

Fear and anxiety were tested in an elevated zero-maze. The apparatus consisted of a circular black Plexiglas runway that was 5-cm wide, 60 cm in diameter and raised 60 cm off of the ground (San Diego Instruments). The runway was divided equally into alternating quadrants of open arcs, enclosed only by a 1 cm lip, and closed arcs, with 25-cm walls. All subjects received one 5-minute trial on two consecutive days, starting in the center of a closed arm, and were recorded by videotracking (EZ Video software, Accuscan Instruments, Columbus, OH). Measures of the total time spent in open and closed arcs, the total number of entries into the open arcs, the latency to enter an open arc and the latency to completely cross an open arc were calculated using the mean of the two trials.

#### Nest building

For small rodents, nests are important for heat conservation, as well as for reproduction and shelter. The mice were initially single-housed in cages that contained no environmental enrichment items, such as bedding, cardboard houses or tunnels. To test their ability to build nests, a paper towel was placed in the home cage 1 hour before the dark phase. The quality of the nest was assessed the next morning using a four-point scale – 0=absence of nest and untouched paper towel, 1=mouse nesting on top of unmodified paper (no enclosing walls), 2=partially shredded paper and shaped nest (enclosing walls), 3=fully shredded paper and shaped nest.

#### Three-chambered social approach test

Sociability and preference for social novelty and social recognition were tested in an automated three-chambered apparatus (Nadler et al., 2004).

The subject mouse was first placed in the central, neutral chamber and allowed to explore for 5 minutes with all doors closed. Next, the doors were opened and the mouse was allowed to freely explore the three empty chambers for an additional 5 minutes. Lack of side preference was confirmed during this habituation. During the testing phases, two empty wire cages – which allow for olfactory, visual, auditory and tactile contacts but not for sexual contact or fighting – containing either an inanimate object (black cone) or a male mouse were placed in each of the testing chambers.

In the test for sociability, the subject was given a choice between spending time with an unfamiliar mouse versus time with an inanimate object. An adult mouse from a different strain that has had no previous contact with the subject was used as an unfamiliar mouse. In the test of preference for social novelty and social recognition, the subject was given a choice between an unfamiliar versus familiar mouse. Another adult mouse from a different strain that had no previous contact with the subject was used as the unfamiliar mouse and the same mouse that was examined in the previous test was used as the familiar mouse.

Each of the tests lasted for 10 minutes and videotracking (EZ Video software) was used for recording the amount of time that the subject spent in each chamber.

#### Y-maze test

The Y-maze alternation is a test of working memory that is based on the natural tendency of mice to explore new territory whenever possible. Mice were placed into the closed bottom arm of a Plexiglas Y-maze (the start arm was 5-cm-wide and 50-cm-long, the choice arms were 5-cm-wide and 15-cm-long, each was set at 135° relative to the start arm) for 5 seconds, then the door was removed to allow access to the choice arms. The subjects were allowed to correct their choice until they entered an arm with all limbs, then the opposing arm was blocked until the subject returned on its own to the start arm. The start arm door was then closed for 5 seconds before starting a new trial. A choice was considered correct when the mouse entered an arm that was different to the one that it had just explored. Subjects were allowed to make as many choices as desired within 10 minutes, but only subjects making at least ten choices were scored.

#### Contextual and cued fear conditioning

To isolate the effects of cued and contextual fear conditioning, a 3-day assay was employed. During the training session, the mice were placed in an ethanol-cleaned contextual box with a bar floor and black-and-white-striped walls, in which all movement could be recorded (Freeze Frame, San Diego Instruments), and given 5 minutes to habituate. Movements were then recorded for 540 seconds. At 120, 260 and 400 seconds after the beginning of the recording, the mice were exposed to a 20-second tone (80 dB, 2 KHz) and a co-terminating shock (1 second, 0.7 mA). At 24 hours after the training phase the animals were tested for contextual memory in the identical enclosure and the movements were recorded for 240 seconds to assess the ability of the animal to remember the context in which the shock had occurred on the previous day. Forty-eight hours after the training phase, the animals were tested for cued memory in a different context (isopropanol-cleaned with a white-wall insert over a mesh grid floor). The mice were recorded for 330 seconds and were then presented with the identical tone from the training session at 120 and 260 seconds after the beginning of the recording session to assess the ability of each animal to remember the tone and to pair it with the shock from training session. Freezing, defined as lack of movement except for respiration, was recorded during each phase.

### Statistical analyses

*Shank3* wild-type, heterozygous and knockout littermates were compared for each parameter. In order to account for possible litter effects, statistical analyses were performed independently for each strain with SPSS 11.0 software using ANCOVA, with or without repeated measures depending on the test. Genotype was taken as an independent variable and the litter as covariate. The analyses were followed by pairwise comparisons when required. The distribution of the genotypes was compared to Mendelian expectation by using Pearson’s chi-square test; the distribution of the nest building score was analyzed using the Kruskal-Wallis test; and the social preference test and the spontaneous alternation test was assessed for each group by using a Student’s *t*-test. The values are expressed as means±s.e.m.

## Supplementary Material

Supplementary Material
