# Supplementary material for: Absence of strong strain effects in behavioral analyses of Shank3-deficient mice
Source: Dis Model Mech. 2014 Mar 20;7(6):667–81. doi: 10.1242/dmm.013821 (PMC4036474; doi:10.1242/dmm.013821)
Supplement: Supplementary Material [file supp_7_6_667__index.html]

Absence of strong strain effects in behavioral analyses of Shank3-deficient mice — Supplementary Material 

# Absence of strong strain effects in behavioral analyses of *Shank3*-deficient mice

## DMM013821 Supplementary Material

**Files in this Data Supplement:**

- **Supplementary Material**
