# Supplementary material for: Absence of strong strain effects in behavioral analyses of Shank3-deficient mice
Source: Dis Model Mech. 2014 Mar 20;7(6):667–81. doi: 10.1242/dmm.013821 (PMC4036474; doi:10.1242/dmm.013821)
Supplement: Supplementary Material [file supp_7.6.667_DMM013821.pdf]

Strain	129							C57							FVB						
Genotype	WT	Het	KO	ANCOVA				WT	Het	KO	ANCOVA				WT	Het	KO	ANCOVA			
n	19	23	12	p	WT/Het	WT/KO	Het/KO	17	20	17	p	WT/Het	WT/KO	Het/KO	18	19	17	p	WT/Het	WT/KO	Het/KO
Blood analysis, chemical																					
Alkaline phosphatase	146.67±3.84	220.00±42.85	155.00±3.21	0.154	-	-	-	127.33±1.20	102.00±9.54	122.33±4.70	0.059	0.062	1.000	0.127	119.67±19.41	132.33±5.84	165.00±3.00	0.173	-	-	-
Alanine aminotransferase	78.33±24.18	82.33±24.26	94.00±31.24	0.913	-	-	-	60.00±12.06	48.67±4.48	47.67±15.67	0.778	-	-	-	44.43±6.64	24.67±1.33	37.33±9.21	0.183	-	-	-
Asparate aminotransferase	116.00±25.03	134.00±37.72	114.67±21.49	0.854	-	-	-	120.00±48.69	134.00±30.86	76.33±9.02	0.493	-	-	-	57.67±27.39	87.67±24.46	68.75±5.81	0.589	-	-	-
Creatine kinase	125.67±27.70	320.00±66.15	207.67±44.85	0.074	0.064	0.491	0.295	258.67v135.18	440.00±173.00	134.67±23.90	0.307	-	-	-	267.67±157.49	235.00±1373.75	100.00±19.00	0.726	-	-	-
albumin	3.00±0.20	3.90±0.90	3.15±0.15	0.560	-	-	-	3.17±0.12	3.00±0.00	3.23±0.07	0.315	-	-	-	3.23±0.05	3.30±0.15	3.33±0.05	0.660	-	-	-
total protein	5.10±0.21	5.93±0.66	5.17±0.17	0.344	-	-	-	5.93±0.24	5.53±0.29	5.87v0.09	0.449	-	-	-	5.93±0.09	5.90±0.15	5.87±0.09	0.919	-	-	-
globulin	2.10±0.06	1.90±0.20	2.10±0.006	0.471	-	-	-	2.73±0.18	2.60±0.21	2.63±0.03	0.831	-	-	-	2.73±0.09	2.75±0.05	2.50±0.00	0.148	-	-	-
Blood urea nitrogen	22.67±2.03	28.33±6.44	20.00±1.53	0.382	-	-	-	26.00±2.08	23.33±2.40	23.67±1.67	0.634	-	-	-	21.67±0.88	21.33±0.33	23.67±0.88	0.134	-	-	-
Cholesterol	92.50±6.50	110.67±26.59	83.00±1.53	0.549	-	-	-	193.00±9.00	180.00±13.58	187.67±4.48	0.699	-	-	-	126.50±0.50	123.00±5.00	121.50±2.50	0.592	-	-	-
Glucose	322.33±19.46	496.00±186.28	270.33±23.24	0.371	-	-	-	371.67±23.40	408.67±60.99	354.00±24.58	0.641	-	-	-	232.00±34.59	258.50±17.68	261.75±6.46	0.566	-	-	-
Calcium	11.00±0.15	14.90±4.06	10.57±0.17	0.411	-	-	-	9.03±0.44	8.77±0.12	8.77±0.50	0.860	-	-	-	10.13±0.97	9.97±0.20	10.80±0.20	0.595	-	-	-
Phosphorus	12.30±0.89	19.23±6.02	13.50±1.31	0.403	-	-	-	12.10±1.30	14.13±0.19	13.13±0.93	0.366	-	-	-	11.87±0.89	10.73±0.55	10.95±0.60	0.520	-	-	-
Total Carbon dioxyde	17.00±0.58	18.67±4.70	18.00±0.58	0.913	-	-	-	17.33±1.76	17.33±2.03	19.33±0.33	0.606	-	-	-	20.50±2.50	18.67±2.33	22.50±2.50	0.584	-	-	-
Albumin Globulin ratio	1.43±0.12	2.13±0.84	1.43±0.07	0.540	-	-	-	1.13±0.03	1.13±0.07	1.23±0.03	0.296	-	-	-	1.17±0.07	1.13±0.09	1.37±0.07	0.134	-	-	-
Hemolysis index	1.00±0.00	1.00±0.58	0.33±0.33	0.422	-	-	-	0.67±0.33	0.67±0.33	0.33±0.33	0.729	-	-	-	1.33±0.67	0.0±0.00	3.67±3.18	0.429	-	-	-
Lipemia index	0.00±0.00	0.00±0.00	0.00±0.00	-	-	-	-	0.00±0.00	0.00±0.00	0.00±0.00	-	-	-	-	0.00±0.00	0.00±0.00	0.00±0.00	-	-	-	-
Blood analysis, complete blood count																					
White blood cells	6.50±1.50	6.07±2.45	6.83±1.79	0.964	-	-	-	5.37±2.19	6.70±0.59	8.13±1.59	0.512	-	-	-	6.10±0.35	8.23±1.28	7.97±1.45	0.407	-	-	-
Red blood cells	8.98±0.12	8.96±0.20	9.07±0.14	0.893	-	-	-	9.48±0.29	9.51±0.16	9.24±0.28	0.713	-	-	-	9.85±0.20	9.55±0.12	9.30v0.15	0.130	-	-	-
hemoglobin	13.95±0.25	13.70±0.31	13.77±0.32	0.865	-	-	-	14.23±0.41	13.73±0.19	13.67v0.55	0.591	-	-	-	15.97±0.33	15.67±0.20	14.00±1.30	0.239	-	-	-
hematocrit	59.35±0.85	58.33±1.93	59.30±0.64	0.846	-	-	-	55.13±1.50	54.70±0.53	54.53±1.57	0.945	-	-	-	63.43±1.40	62.03±0.69	60.40±0.26	0.141	-	-	-
Mean corpuscular volume	66.00±00	65.00±1.00	65.33±0.33	0.659	-	-	-	58.33±2.33	57.67±0.67	59.00±0.00	0.804	-	-	-	64.33±0.33	65.00±0.00	65.00±1.00	0.687	-	-	-
mean corpuscular hemoglobin	15.55±0.05	15.27±0.03	15.20±0.10	0.055	0.104	0.053	0.784	15.03±0.22	14.47±0.12	14.80±0.15	0.135	-	-	-	16.20±0.06	16.43±0.03	15.10±1.60	0.584	-	-	-
mean corpuscular hemoglobin concentration	23.55±0.05	23.47±0.37	23.20±0.26	0.718	-	-	-	25.87±1.27	25.13±0.20	25.03±0.32	0.715	-	-	-	25.20±0.10	25.30±0.06	23.23±2.22	0.482	-	-	-
Nucleated red blood cells for 100WBC	0.00±0.00	0.00±0.00	0.00±0.00	-	-	-	-	0.00±0.00	0.00±0.00	0.00±0.00	-	-	-	-	0.00±0.00	0.00±0.00	0.00±0.00	-	-	-	-
Neutrophil	19.20±0.50	21.67±8.35	28.80±10.59	0.753	-	-	-	19.23±00	9.43±1.98	10.33±0.18	0.381	-	-	-	13.80±2.00	10.57±0.60	12.00±1.06	0.309	-	-	-
Lymphocyte	73.45±0.25	71.53±9.02	66.97±10.93	0.890	-	-	-	82.50±3.07	87.23±2.03	85.13±0.88	0.370	-	-	-	78.87±4.95	83.00±1.31	82.20±0.67	0.609	-	-	-
Monocyte	2.05±0.45	2.80±0.62	1.67±0.63	0.444	-	-	-	3.50±2.25	0.77±0.15	1.03±0.03	0.332	-	-	-	2.03±0.32	1.20±0.10	1.77±0.63	0.403	-	-	-
Eosinophil	3.50±0.30	2.60±2.03	1.20±0.10	0.548	-	-	-	1.40±1.11	0.63±0.15	1.53±0.58	0.662	-	-	-	4.40±3.11	3.73±1.05	2.90±0.59	0.862	-	-	-
Basophil	0.30±0.00	0.43±0.30	0.27±0.03	0.815	-	-	-	0.13±0.009	0.33±0.03	0.20±0.06	0.158	-	-	-	0.20±0.12	0.30±0.00	0.17±0.09	0.540	-	-	-
Platelet estimate	0.50±0.50	-0.33±0.33	0.67±0.33	0.199	-	-	-	0.33±0.33	0.67±0.33	0.67±0.33	0.729	-	-	-	0.00±0.00	0.00±0.00	0.00±0.00	-	-	-	-
Asolute neutrophil	1240.5±255.5	1352.0±943.3	2262.6±1304.4	0.770	-	-	-	661.0±190.9	613.0±106.0	834.6±149.5	0.587	-	-	-	831.7±99.4	884.3±174.2	969.3v209.4	0.846	-	-	-
Absolute lymphocyte	4778.0±1118.0	4407.0±1878.4	4275.3±595.8	0.970	-	-	-	4490.3±1874.8	5863.3±635.5	6897.6±1279.3	0.497	-	-	-	4814.0±439.2	6804.67±979.19	6538.3±1176.2	0.321	-	-	-
Absolute Monocyte	140.00±60.00	189.33±87.57	123.33±63.28	0.807	-	-	-	89.33±10.35	49.67±5.61	83.00±14.47	0.082	0.089	0.910	0.152	123.33±18.02	101.33±22.26	147.00±58.04	0.703	-	-	-
Absolute Eosinophil	223.00±33.00	62.33±32.27	80.33±17.89	0.025	-	-	-	103.33±80.98	41.67±8.74	141.33±76.89	0.576	-	-	-	274.00±188.00	315.33±121.83	218.67±32.50	0.874	-	-	-
Absolute Basophil	19.50±4.50	13.00±7.81	16.67±6.23	0.776	-	-	-	10.00±6.81	22.67±4.26	18.00±8.14	0.441	-	-	-	12.33±6.94	24.67±3.84	12.33±6.33	0.301	-	-	-
Percentage reticulocyte	2.90±0.20	0.30±0.10	1.10±0.10	0.002**	0.002**	0.006**	0.056	1.15±0.35	-	0.60±0.20	0.306	-	-	-	3.27±2.12	4.20±2.10	3.75±0.55	0.944	-	-	-
Absolute reticulocyte	260.50±14.50	27.50±9.50	101.00±11.00	0.002**	0.002**	0.005**	0.044*	106.00±28.00	-	54.50v15.50	0.249	-	-	-	318.33±204.33	3.99±2.03	344.50±55.50	0.955	-	-	-
Platelet count	1493.0±142.0	1058.0±358.0	1424.0±212.6	0.546	-	-	-	1538.5±191.5	1698.3±155.8	1662.6±221.8	0.859	-	-	-	992.7±139.8	808.33±330.33	1107.6±65.7	0.622	-	-	-

Supplementary table 1: Complete blood count and blood chemistry screen in Shank3-deficient mice.

Strain	129							C57							FVB						
Genotype	WT	Het	KO	ANCOVA				WT	Het	KO	ANCOVA				WT	Het	KO	ANCOVA			
n	19	23	12	p	WT/Het	WT/KO	Het/KO	17	20	17	p	WT/Het	WT/KO	Het/KO	18	19	17	p	WT/Het	WT/KO	Het/KO
Tail flick																					
Latency to flick, trial 1 (sec)	6.60 ± 0.34	7.36±0.66	6.42±0.16	0.488	-	-	-	6.06±0.53	6.51±0.42	8.31±0.73	0.021*	0.579	0.009**	0.028*	9.58±0.63	9.99±0.30	9.64±0.41	0.735	-	-	-
Latency to flick, trial 2 (sec)	6.50 ± 0.34	7.34±0.64	7.17+0.43	0.499	-	-	-	67.21±0.64	7.68±0.66	6.91±0.45	0.709	-	-	-	10.04±0.50	9.33±0.52	10.55±0.34	0.180	-	-	-
Latency to flick, trial 3 (sec)	7.46 ± 0.42	7.06±0.70	8.12±0.61	0.455	-	-	-	7.12±0.59	7.51±0.68	7.31±0,48	0.904	-	-	-	9.85±0.46	9.03±0.53	9.42±0.52	0.573	-	-	-
Latency to flick, mean(sec)	6.86±0.23	7.27±0.60	7.24±0.26	0.772	-	-	-	6.80±0.36	7.23±0.39	7.53±0.40	0.407	-	-	-	9.83±0.43	9.45±0.32	9.87±0.29	0.663	-	-	-
Grip strength																					
Best (gm)	137.26±3.11	140.22±2.54	130.58±3.61	0.153	-	-	-	158.24±4.08	147.40±4.41	157.61±3.74	0.111	-	-	-	158.63±3.87	148.65±5.22	156.33±6.66	0.431	-	-	-
Mean (gm)	127.86±2.87	128.84±2.13	119.72±3.46	0.097	0.790	0.070	0.039	143.33±3.33	135.90±3.35	145.67±3.75	0.114	-	-	-	140.51±3.80	138.04±5.03	144.69±6.26	0.673	-	-	-
Rotarod																					
Latency to fall, trial 1 (sec)	21.00±2.53	16.83±1.58	23.42±2.68	0.147	-	-	-	30.65±2.98	35.60±3.60	32.94±3.49	0.582	-	-	-	17.82±1.93	17.89±2.22	21.44±1.85	0.545	-	-	-
Latency to fall, trial 2 (sec)	18.58±3.03	18.47±1.66	23.25±3.17	0.275	-	-	-	40.47±2.75	28.40±3.33	44.94±3.60	0.368	-	-	-	17.59±2.23	24.47±2.85	21.44±2.35	0.158	-	-	-
Latency to fall, trial 3 (sec)	15.96±1.25	22.39±2.19	24.17±4.45	0.056	0.059	0.030	0.517	40.06±4.40	39.05±3.33	38.21±4.88	0.952	-	-	-	21.41±3.39	26.47±3.49	26.28±2.96	0.592	-	-	-
Latency to fall, best trial (sec)	25.47±2.97	25.22±2.02	29.75±3.85	0.473	-	-	-	47.47±3.18	47.85±2.53	49.29±3.34	0.915	-	-	-	24.12±2.97	31.47±3.46	29.06±2.68	0.257	-	-	-
Latency to fall, mean (sec)	18.51±1.70	19.23±1.42	23.61±2.86	0.146	-	-	-	37.06±2.76	37.68±2.24	38.70±3.26	0.923	-	-	-	18.94±2.22	22.95±2.32	23.06±2.00	0.457	-	-	-
Beam walking																					
Latency to start (sec)	15.47±3.21	17.76±2.83	14.24±3.35	0.856	-	-	-	9.77±2.42	6.56±1.55	3.19±0.92	0.036*	0.178	0.010**	0.176	11.48±2.45	11.73±2.64	14.47±3.09	0.501	-	-	-
Distance (cm)	39.46±2.06	30.23±2.42	40.09±2.58	0.006**	0.004**	0.927	0.020*	42.92±1.74	43.78±1.35	43.26±1.60	0.893	-	-	-	41.14±1.92	41.72±1.78	39.25±2.05	0.664	-	-	-
Crossing time (sec)	24.85±3.18	24.51±3.60	16.29±2.14	0.232	-	-	-	15.08±1.83	13.08±0.89	9.78±0.73	0.013*	0.234	0.003**	0.050*	18.13±1.50	14.93±1.20	14.70±1.35	0.253	-	-	-
Speed (cm/sec)	3.00±0.22	3.80±0.39	4.52±0.45	0.022**	0.083	0.008**	0.192	4.31±0.25	4.90±0.31	6.30±0.37	<0.001***	0.345	<0.001***	<0.001***	3.62±0.26	4.76±0.45	4.39±0.34	0.137	-	-	-
% partial cross (freezing)	11.59±3.88	22.50±4.70	11.11±4.74	0.072	0.073	0.563	0.048	7.58±3.28	2.53±1.78	1.47±1.47	0.142	-	-	-	9.86±3.56	5.63±2.76	7.35±3.19	0.661	-	-	-
% falls	23.19±5.12	41.25±5.54	20.00±6.03	0.037*	0.015*	0.805	0.103	9.09±3.57	12.50±3.72	10.29v3.71	0.825	-	-	-	18.31±4.62	14.08±4.16	22.06±5.07	0.503	-	-	-
% of false starts	4.35±2.47	1.25±1.25	4.44±3.11	0.516	-	-	-	3.03±2.13	3.75±2.14	1.14±1.47	0.756	-	-	-	2.82±1.98	0.00±0.00	1.47±1.47	0.403	-	-	-
Number of slips	2.64±0.26	2.20±0.19	2.67±0.27	0.331	-	-	-	2.56±0.36	2.46±0.20	2.63±0.19	0.899	-	-	-	0.69±0.10	0.75±0.09	0.93±0.12	0.165	-	-	-
Open field locomotion																					
Moving distance 1hr (cm)	3995.6±341.3	4357.7±248.5	5171.83±385.27	0.127	-	-	-	7373.1±468.92	7242.2±567.8	6870.4±673.4	0.827	-	-	-	12446.9±1007.6	12837.0±747.3	13394.5±533.7	0.633	-	-	-
Moving distance time x genotype effect	-	-	-	0.009**	0.367	0.043*	0.181	-	-	-	0.845	-	-	-	-	-	-	0.290	-	-	-
Moving distance 0-10 min (cm)	1279.7±109.0	1627.9±80.7	1694.9±80.7	0.014*	0.011*	0.015*	0.739	2026.5±146.4	1976.5±179.0	1874.6±187.1	0.831	-	-	-	2974.7±155.9	3154.6±151.4	3281.5±168.7	0.317	-	-	-
Moving distance 10-20 min (cm)	657.11±71.17	764.09±51.85	764.09±51.82	0.158	-	-	-	1182.8±87.8	1143.9±97.6	1080.8±121.5	0.792	-	-	-	2106.1±148.1	2213.6±135.0	2281.8±120.7	0.544	-	-	-
Moving distance 20-30 min (cm)	715.33±88.94	694.77±58.14	697.77±58.14	0.773	-	-	-	1175.0±86.3	1061.8±72.8	1056.6±109.2	0.555	-	-	-	1887.1±174.4	2057.2±131.5	2139.3±93.4	0.313	-	-	-
Moving distance 30-40 min (cm)	563.83±64.23	477.23±51.75	477.23±51.75	0.150	-	-	-	1120.6±76.0	1110.2±110.7	1007.3±108.3	0.693	-	-	-	1889.28±157.90	1956.32±131.50	2028.5±105.6	0.689	-	-	-
Moving distance 40-50 min 5 (cm)	458.06±51.21	436.77±66.77	436.77±66.77	0.475	-	-	-	1000.2±84.7	989.21±76.12	1032.9±118.0	0.946	-	-	-	1815.3±194.9	1735.7±133.4	1886.4±83.5	0.774	-	-	-
Moving distance 50-60 min (cm)	321.5±54.32	356.95±42.56	356.95±42.56	0.213	-	-	-	867.94±81.94	960.53±102.27	818.25±92.31	0.557	-	-	-	1774.4±234.3	1719.9±120.4	1776.8±95.5	0.964	-	-	-
Moving time 1hr (sec)	509.81±41.37	553.32±31.79	638.63±34.94	0.129	-	-	-	746.64±38.26	758.14±43.46	720.41±54.71	0.835	-	-	-	1014.0±61.9	1034.4±45.0	1058.9±38.6	0.813	-	-	-
Moving time time x genotype effect	-	-	-	0.017*	0.737	0.044*	0.180	-	-	-	0.450	-	-	-	-	-	-	0.225	-	-	-
Moving time 0-10 min (sec)	155.36±12.34	194.32±8.45	198.32±8.95	0.008**	0.007**	0.009**	0.678	190.57±8.29	192.22±10.86	176.51±12.87	0.546	-	-	-	222.63±7.78	230.67±7.91	240.94±10.28	0.304	-	-	-
Moving time 10-20 min (sec)	82.48±8.92	96.95±6.39	110.95±9.28	0.127	-	-	-	121.82±8.24	124.34±7.50	115.21±8.93	0.749	-	-	-	173.38±8.66	177.82±8.59	184.31±7.60	0.638	-	-	-
Moving time 20-30 min (sec)	90.60±10.18	91.34±7.71	103.83±8.42	0.828	-	-	-	119.07±6.70	113.47±6.43	114.97±9.62	0.840	-	-	-	155.46±11.22	170.50±9.39	171.52±6.21	0.298	-	-	-
Moving time 30-40 min (sec)	75.34±8.39	64.34±7.71	84.28±6.94	0.287	-	-	-	116.79±7.10	115.71±9.17	110.30±8.71	0.848	-	-	-	158.54±10.27	162.28±8.38	161.90±7.93	0.936	-	-	-
Moving time 40-50 min (sec)	61.84±6.73	47.15±8.47	73.53±8.21	0.393	-	-	-	107.21±8.21	107.39±7.15	112.33±10.47	0.901	-	-	-	153.67±14.92	145.48±9.29	152.25±7.48	0.788	-	-	-
Moving time 50-60 min (sec)	44.18±7.26	48.59±5.77	67.73±12.05	0.212	-	-	-	91.18±8.21	105.00±9.58	91.09±7.50	0.414	-	-	-	150.32±14.89	147.69±8.03	147.96±8.91	0.978	-	-	-

Supplementary table 2: Sensory-motor performances in *Shank3*-deficient mice.

Strain	129							C57							FVB						
Genotype	WT	Het	KO	ANCOVA				WT	Het	KO	ANCOVA				WT	Het	KO	ANCOVA			
n	19	23	12	p	WT/Het	WT/KO	Het/KO	17	20	17	p	WT/Het	WT/KO	Het/KO	18	19	17	p	WT/Het	WT/KO	Het/KO
Open field thygmotaxis																					
Center distance 1hr (cm)	1301.9±169.4	1084.0±136.8	1276.8±213.2	0.564	-	-	-	2554.6±295.5	2044.2±185.5	1759.7±216.1	0.060	0.125	0.020	0.354	4390.1±322.7	4919.4±354.2	4764.6±307.5	0.582	-	-	-
Center distance time x genotype effect	-	-	-	0.522	-	-	-	-	-	-	0.858	-	-	-	-	-	-	0.199	-	-	-
Center time 1hr (sec)	678.29±118.15	389.15±46.58	667.59±122.21	0.047*	0.024*	0.923	0.068	836.91±83.45	832.46±87.04	719.93±90.36	0.552	-	-	-	669.05±51.59	689.89±39.93	670.88±48.30	0.949	-	-	-
Center time time x genotype effect	-	-	-	0.063	-	-	-	-	-	-	0.006**	1.000	0.349	0.337	-	-	-	0.110	-	-	-
Number of center entries 1hr	122.89±14.55	101.91±11.27	122.75±19.38	0.516	-	-	-	240.47±23.86	196.16±17.20	176.94±21.65	0.099	0.136	0.037	0.480	362.89±23.26	414.47±23.52	384.06±24.28	0.345	-	-	-
Center entries time x genotype effect	-	-	-	0.615	-	-	-	-	-	-	0.773	-	-	-	-	-	-	0.484	-	-	-
Center rest 1hr (sec)	518.22±100.51	262.86±33.10	528.92±113.45	0.036*	0.020*	0.999	0.049*	590.26±67.81	618.49±74.81	531.30±78.05	0.676	-	-	-	365.02±36.11	356.12±30.33	350.98±34.02	0.944	-	-	-
Center rest time x genotype effect	-	-	-	0.060	-	-	-	-	-	-	0.006**	0.757	0.557	0.382	-	-	-	0.149	-	-	-
Center rest 0-10 min (sec)	63.86±17.32	51.86±11.33	88.32±36.04	0.843	-	-	-	41.50±5.90	80.71±13.84	73.77±12.92	0.050*	0.021*	0.063	0.690	30.86±3.29	33.34±4.34	27.71±3.63	0.602	-	-	-
Center rest 10-20 min (sec)	76.69±17.98	46.85±12.42	58.53±20.37	0.394	-	-	-	82.65±12.81	92.28±13.18	81.26±11.88	0.793	-	-	-	51.66±5.76	65.15±6.12	52.71±3.63	0.275	-	-	-
Center rest 20-30 min (sec)	106.24±22.06	45.42±9.19	70.46±22.71	0.033*	0.012*	0.085	0.690	102.14±16.57	83.37v13.45	86.68±16.70	0.668	-	-	-	62.96±9.05	63.36±7.09	70.48±9.71	0.774	-	-	-
Center rest 30-40 min (sec)	78.84±20.40	37.45±9.19	83.98±22.26	0.071	0.059	0.711	0.050	127.55±13.94	104.83±19.30	101.28±18.57	0.510	-	-	-	69.20±8.55	60.80±7.27	62.09±7.40	0.648	-	-	-
Center rest 40-50 min (sec)	90.16±22.59	35.88±9.35	98.58±25.64	0.019*	0.025*	0.544	0.013*	119.72±18.80	118.81±16.22	111.86±22.02	0.954	-	-	-	65.52±8.25	61.42±7.98	68.88±8.39	0.818	-	-	-
Center rest 50-60 min (sec)	102.44±23.80	45.40±9.83	129.05±32.85	0.020*	0.042*	0.367	0.010**	116.71±19.98	140.49±18.74	76.46±16.74	0.039	0.319	0.119	0.012	84.83±11.00	72.77±6.38	69.82±8.01	0.368	-	-	-
Open field vertical activity																					
Number of rears 1hr	52.17±12.69	52.00±9.61	43.92±12.70	0.971	-	-	-	414.47±22.23	396.05±15.62	337.00±23.78	0.049*	0.568	0.021*	0.065	696.11±20.55	709.79±20.52	776.24±30.87	0.050*	0.654	0.025*	0.058
Number of rears time x genotype effect	-	-	-	0.849	-	-	-	-	-	-	0.207	-	-	-	-	-	-	0.702	-	-	-
Elevated zero maze																					
Total moving time (sec)	78.58±5.76	88.00±4.44	78.85±4.81	0.415	-	-	-	93.90±4.60	103.03±5.79	121.04±8.46	0.018*	0.439	0.006**	0.030*	113.25±7.01	129.40±6.87	153.86±7.98	0.002**	0.344	0.001***	0.009**
Total distance (cm)	1636.7±116.6	1884.6±101.8	1818.2±113.3	0.333	-	-	-	1404.7±75.4	1537.6±83.7	1977.0±182.8	0.002**	0.532	0.001***	0.003**	1864.4±182.1	2250.2±157.7	2807.7±196.1	0.003**	0.372	0.001***	0.010**
Time in open arm (sec)	113.99±8.41	110.64±4.40	100.95±7.77	0.612	-	-	-	72.85±5.74	88.76±5.76	95.93±6.74	0.027*	0.079	0.008**	0.262	120.13±6.14	131.97±4.54	153.12±8.09	0.007**	0.204	0.002**	0.040*
Time in closed arm (sec)	186.01±8.41	189.36±4.40	199.05±7.77	0.612	-	-	-	227.15±5.74	211.24±5.76	204.07±6.74	0.027*	0.079	0.008**	0.262	179.87±6.14	168.03±4.54	146.89±8.09	0.007**	0.204	0.002**	0.040*
Ratio Open/close	0.72±0.09	0.63±0.04	0.56±0.08	0.427	-	-	-	0.34±0.04	0.46±0.04	0.53±0.06	0.018*	0.096	0.005**	0.157	0.75±0.07	0.86±0.05	1.20±0.12	0.002**	0.355	0.001***	0.008**
Latency to enter open arm (sec)	13.66±3.78	12.16±1.89	9.07±3.21	0.698	-	-	-	23.09±5.69	17.41±3.31	14.05±3.84	0.303	-	-	-	18.18±5.16	8.21±1.25	7.80±1.42	0.065	0.043	0.040	0.928
Number of entries in open arm	26.37±1.57	29.98±1.31	32.00±2.01	0.050*	0.081	0.023*	0.416	21.88±1.27	23.75±1.35	28.50±2.27	0.008**	0.532	0.004**	0.012*	32.44±2.36	35.55±1.73	40.21±2.07	0.083	0.671	0.037	0.080

Supplementary table 3: Anxiety-like behavior in *Shank3*-deficient mice.

Strain	129							C57							FVB						
Genotype	WT	Het	KO	T-test, p-value				WT	Het	KO	T-test, p-value				WT	Het	KO	T-test, p-value			
n	19	23	12	Test	WT	Het	KO	17	20	17	Test	WT	Het	KO	18	19	17	Test	WT	Het	KO
3 chambered social interaction test - sociability																					
time exploring mouse (sec)	210.53±26.22	200.61±15.98	219.08±17.48	mouse vs object	0.028*	<0.001***	0.001***	139.65±8.62	134.10±7.86	165.35±12.27	mouse vs object	0.003**	0.007**	<0.001***	220.72±13.26	214.10±10.55	276.23±22.54	mouse vs object	<0.001***	<0.001***	<0.001***
time exploring object (sec)	103.10±21.65	84.78±15.426	121.00±11.40					91.29±7.74	98.75±6.65	85.85±5.60					8.983±8.77	87.37±6.89	81.59±12.44				
3 chambered social interaction test - preference for social novelty and social recognition																					
time exploring familiar mouse (sec)	121.00±25.20	102.26±13.83	103.75±21.57	familiar vs unfamiliar	0.210	0.130	0.071	73.00±6.46	67.85±6.27	79.41±7.51	familiar vs unfamiliar	<0.001***	<0.001***	<0.001***	107.22±10.78	99.89±8.02	104.88±12.79	familiar vs unfamiliar	0.001***	0.001***	0.001***
time exploring unfamiliar mouse (sec)	179.21±25.74	145.35±18.95	184.83±26.10					127.18±9.67	132.70±8.86	156.06±12.11					167.39±10.42	168.95±13.55	232.00±22.87				

Strain	129							C57							FVB						
Genotype	WT	Het	KO	ANCOVA				WT	Het	KO	ANCOVA				WT	Het	KO	ANCOVA			
n	19	23	12	p	WT/Het	WT/KO	Het/KO	17	20	17	p	WT/Het	WT/KO	Het/KO	18	19	17	p	WT/Het	WT/KO	Het/KO
Nest building																					
Nest building, mean score	2.26±0.10	2.48±0.12	2.42±0.19	0.417	-	-	-	2.23±0.25	2.55±0.21	2.18±0.27	0.519	-	-	-	1.89±0.16	1.59±0.14	1.76±0.16	0.266	-	-	-

Supplementary table 4: Social behavior in *Shank3*-deficient mice

Strain	129							C57							FVB						
Genotype	WT	Het	KO	ANCOVA				WT	Het	KO	ANCOVA				WT	Het	KO	ANCOVA			
n	19/14*	23/19*	12/9*	p	WT/Het	WT/KO	Het/KO	17/16*	20/20*	17/14*	p	WT/Het	WT/KO	Het/KO	18/12*	19/14*	17/12*	p	WT/Het	WT/KO	Het/KO
Fear conditioning																					
Day1, pre-shock, 0 to 120 sec (% freezing)	0.31±0.14	0.18±0.08	0.17±0.09	0.589	-	-	-	0.21±0.11	0.07±0.05	0.18±0.18	0.664	-	-	-	0.00±0.00	0.00±0.00	0.00±0.00	-	-	-	-
Day1, tone 1, 120 to 140 sec (% freezing)	0.98±0.68	0.23±0.23	0.00±0.00	0.321	-	-	-	1.26±0.69	0.47±0.22	0.00±0.00	0.109	-	-	-	0.07±0.07	0.28±0.19	0.31±0.21	0.753	-	-	-
Day1, post-shock 1, 140 to 260 (% freezing)	8.50±1.76	2.85v0.85	2.03±0.82	0.002**	0.002**	0.002**	0.583	5.25±1.37	3.49±0.96	2.83±0.90	0.295	-	-	-	0.03±0.03	0.09±0.05	0.05±0.04	0.556	-	-	-
Day1, tone 2, 260 to 280 (% freezing)	11.74±2.98	11.69±3.23	5.18±2.19	0.361	-	-	-	7.71±2.16	3.58±1.61	4.85±1.78	0.284	-	-	-	0.00±0.00	0.00±0.00	0.00±0.00	-	-	-	-
Day1, post-shock 2, 280 to 400 sec (% freezing)	45.33±3.61	28.93±3.92	15.39±4.78	<0.001***	0.003**	<0.001***	0.065	18.18±2.93	14.12±3.09	13.94±2.98	0.552	-	-	-	0.15±0.08	0.20±0.09	0.00±0.00	0.093	0.675	0.103	0.037
Day1, tone 3, 400 to 420 sec (% freezing)	56.33±3.61	43.90±5.42	18.74±6.37	0.002**	0.086	<0.001***	0.020*	23.23±4.18	19.93±3.12	21.98±4.61	0.830	-	-	-	0.30±0.23	0.21±0.16	0.31±0.24	0.909	-	-	-
Day1, post-shock 3, 420 to 540 sec (% freezing)	46.88±4.66	33.81±4.28	14.24±2.95	<0.001***	0.031*	<0.001***	0.001***	20.97±2.89	12.00±2.59	18.98±2.62	0.052	0.022	0.598	0.075	0.58±0.27	0.42±0.23	0.10±0.10	0.163	-	-	-
Day2, 0 to 60 sec (% freezing)	13.20±2.97	11.31±2.66	14.19±2.97	0.748	-	-	-	8.48±2.53	6.29±1.50	8.55±2.64	0.701	-	-	-	0.60±0.36	0.52±0.36	0.18±0.13	0.586	-	-	-
Day2, 60 to 120 sec (% freezing)	28.01±3.82	26.97±3.27	27.44±4.06	0.978	-	-	-	15.30±3.05	13.60±3.11	15.99±3.30	0.868	-	-	-	1.08±0.56	0.86±0.42	0.11±0.11	0.273	-	-	-
Day2, 120 to 180 sec (% freezing)	40.08±4.49	36.71±4.29	30.94±3.99	0.503	-	-	-	21.42±3.52	17.92±3.41	18.14±4.31	0.766	-	-	-	1.11±0.66	0.87±0.67	0.00±0.00	0.425	-	-	-
Day2, 180 to 240 sec (% freezing)	42.05±4.40	42.67±4.76	32.85±4.15	0.298	-	-	-	20.57±4.42	19.42±3.62	24.49±4.09	0.668	-	-	-	1.44±0.79	0.97±0.55	0.21±0.14	0.385	-	-	-
Day2, total (% freezing)	30.84±2.94	29.41±2.90	105.42±10.51	0.634	-	-	-	16.94±2.82	14.30±2.58	16.79±3.29	0.810	-	-	-	1.05±0.40	0.80±0.46	0.12±0.05	0.234	-	-	-
Day3, pre-tone, 0 to 60 sec (% freezing)	2.94±0.90	3.79±1.60	6.94±2.36	0.322	-	-	-	4.76±2.12	5.68±2.00	6.92±3.07	0.833	-	-	-	0.10±0.10	0.31±0.19	0.16±0.16	0.491	-	-	-
Day3, pre-tone 60 to 120 sec (% freezing)	17.12±3.86	12.49±2.82	15.36±4.24	0.487	-	-	-	11.48±3.33	10.75±2.15	11.62±3.16	0.980	-	-	-	0.50±0.42	0.35±0.21	0.16±0.11	0.783	-	-	-
Day3, tone 1, 120 to 140 sec (% freezing)	65.30±4.07	53.43±3.67	59.52±5.59	0.116	-	-	-	53.54±5.84	61.51±4.07	49.86±5.59	0.267	-	-	-	18.63±3.76	13.69±3.53	12.51±3.61	0.555	-	-	-
Day3, post-tone 1, 140 to 200 sec (% freezing)	64.28±4.45	53.72±3.72	65.26±5.31	0.089	0.067	0.767	0.065	38.02±6.09	41.82±5.32	43.01±5.22	0.815	-	-	-	6.27±1.35	1.98±0.70	1.71±0.62	0.004**	0.004**	0.004**	0.884
Day3, post-tone 1, 200 to 260 (% freezing)	57.30±5.75	50.62±4.76	58.76±3.56	0.436	-	-	-	28.14±6.07	27.72±3.34	28.91±6.15	0.873	-	-	-	0.70±0.53	0.84±0.70	0.31±0.18	0.793	-	-	-
Day3, post-tone 1, 260 to 290 sec (% freezing)	55.30±5.31	43.50±6.06	52.90±6.77	0.238	-	-	-	28.89±6.20	24.08±5.45	28.33±5.91	0.820	-	-	-	0.84±0.66	1.78±0.90	1.00±0.70	0.588	-	-	-
Day3, tone 2, 290 to 310 sec (% freezing)	78.61±3.80	73.12±4.67	65.37±7.99	0.224	-	-	-	68.61±5.81	61.71±3.67	54.54±4.00	0.116	-	-	-	26.05±4.77	17.81±3.57	20.11±4.39	0.234	-	-	-
Day3, post-tone 2, 310 to 333 sec (% freezing)	789.30±5.25	78.44±3.65	82.09±5.09	0.898	-	-	-	53.10±7.13	53.99±5.96	50.08±5.84	0.904	-	-	-	7.06±2.58	6.05±2.39	1.99±1.35	0.291	-	-	-
Y-maze spontaneous alternation test																					
Total number of choices	10.79±1.30	11.91±0.91	9.75±0.82	0.585	-	-	-	13.53±1.35	15.10±0.96	15.18±1.65	0.619	-	-	-	11.17±2.08	15.47±2.64	14.06±2.76	0.696	-	-	-
Number of correct choice	6.16±0.73	6.65±0.68	5.08±0.89	0.516	-	-	-	8.88±0.77	9.70±0.73	10.71±1.08	0.364	-	-	-	7.00±1.36	8.11±1.54	8.35±1.69	0.978	-	-	-
Correct/total, animals with at least 10 choices	0.58±0.04	0.56±0.05	0.55±0.06	0.927	-	-	-	0.68±0.04	0.65±0.03	0.71±0.03	0.560	-	-	-	0.60±0.04	0.50±0.05	0.60±0.04	0.176	-	-	-

* X/Y, X=total number of mice tested, Y=number of animals that made at least 10 choices

Supplementary table 5: Learning and memory in Shank3-deficient mice

Strain	129							C57							FVB						
Genotype	WT	Het	KO	ANCOVA				WT	Het	KO	ANCOVA				WT	Het	KO	ANCOVA			
n	19	23	12	p	WT/Het	WT/KO	Het/KO	17	20	17	p	WT/Het	WT/KO	Het/KO	18	19	17	p	WT/Het	WT/KO	Het/KO
Revolutions																					
total number of revolution, 1hr	26.39±2.58	29.14±2.18	37.17±3.50	0.091	0.401	0.030	0.121	49.76±3.12	50.42±3.78	47.75±4.63	0.892				79.89±6.15	84.75±5.92	86.71±3.04	0.547	-	-	-
Revolutions time x genotype effect	-	-	-	0.518	-	-	-	-	-	-	0.574	-	-	-	-	-	-	-	-	-	-
Clockwise revolution, 1hr (%)	48.16±3.47	48.36±4.15	56.17±7.02	0.631	-	-	-	53.88±2.84	54.82±3.72	55.46±3.56	0.950	-	-	-	54.36±2.58	51.80±2.70	57.40±3.05	0.384	-	-	-
Revolutions time x genotype effect	-	-	-	0.074	0.410	0.036	0.139	-	-	-	0.629	-	-	-	-	-	-	0.142	-	-	-
Perseverance																					
Perseveration, animals with at least 10 choices	1.21±0.26	1.26±0.23	1.00±0.24	0.907	-	-	-	0.690.22	1.100.25	0.860.29	0.549	-	-	-	1.07±0.35	2.36±0.41	1.33±0.39	0.104	-	-	-
Grooming																					
Latency to first grooming bout (sec)	383.32±36.84	336.39±25.24	359.39±25.24	0.517	-	-	-	171.35±23.60	199.20±25.26	166.76±18.37	0.552	-	-	-	246.61±24.27	249.00±25.59	251.76±35.20	0.939	-	-	-
Total number of bouts	1.53±0.27	1.83±0.18	2.00±0.43	0.579	-	-	-	3.24±0.42	3.65±0.56	2.82±0.27	0.418	-	-	-	2.22±0.32	2.00±.0.22	2.47±0.32	0.506	-	-	-
Total time of grooming (sec)	10.84±1.83	13.57±1.73	15.17±2.44	0.326	-	-	-	18.71±3.95	21.20±2.41	22.47±4.77	0.780	-	-	-	11.78±1.86	10.16±1.54	11.41±1.55	0.785	-	-	-
Barbering																					
n	29	30	18	p	WT/Het	WT/KO	Het/KO	31	41	35	p	WT/Het	WT/KO	Het/KO	35	35	35	p	WT/Het	WT/KO	Het/KO
Percentage of mice with barbering	58.62±9.31	63.33±8.95	83.33±9.04	0.206	-	-	-	0.00±0.00	2.44±2.44	2.86±2.86	0.661	-	-	-	0.00±0.00	0.00±0.00	0.00±0.00	-	-	-	-

Supplementary table 6: Stereotypies, repetitive behavior and perseverance in Shank3-deficient mice.
